# Structural basis for TRIM72 oligomerization during membrane damage repair

**DOI:** 10.1038/s41467-023-37198-1

**Published:** 2023-03-21

**Authors:** Yuemin Ma, Lei Ding, Zhenhai Li, Chun Zhou

**Affiliations:** 1 School of Public Health, and Department of Pathology of Sir Run Run Shaw Hospital, Zhejiang University School of Medicine, Hangzhou, Zhejiang 310058 China; 2 Shanghai Key Laboratory of Mechanics in Energy Engineering, Shanghai Institute of Applied Mathematics and Mechanics, School of Mechanics and Engineering Science, Shanghai University, Shanghai, 200072 China

**Keywords:** X-ray crystallography, Membrane fusion, X-ray crystallography, Membrane structure and assembly

## Abstract

Tripartite Motif Protein 72 (TRIM72, also named MG53) mediates membrane damage repair through membrane fusion and exocytosis. During injury, TRIM72 molecules form intermolecular disulfide bonds in response to the oxidative environment and TRIM72 oligomers are proposed to connect vesicles to the plasma membrane and promote membrane fusion in conjunction with other partners like dysferlin and caveolin. However, the detailed mechanism of TRIM72 oligomerization and action remains unclear. Here we present the crystal structure of TRIM72 B-box-coiled-coil-SPRY domains (BCC-SPRY), revealing the molecular basis of TRIM72 oligomerization, which is closely linked to disulfide bond formation. Through structure-guided mutagenesis, we have identified and characterized key residues that are important for the membrane repair function of TRIM72. Our results also demonstrate that TRIM72 interacts with several kinds of negatively charged lipids in addition to phosphatidylserine. Our work provides a structural foundation for further mechanistic studies as well as the clinical application of TRIM72.

## Abstract (web summary)

TRIM72 oligomerization via intermolecular disulfide bonds is important for cells to repair damaged membranes. Here, authors report the crystal structure of TRIM72 dimer and show that TRIM72 B-box dimerization facilitates TRIM72 oligomerization.

## Introduction

Tripartite motif proteins form a family of more than 80 members encoded in the human genome and participate in a variety of cellular processes, including proliferation, differentiation, apoptosis, autophagy, tumorigenesis, innate immunity, and viral replication^1–3^. TRIM proteins usually contain a RING finger domain followed by either one or two B-box domains and a coiled­-coil domain in the N-terminus^4^. Both RING and B-box domains contain conserved zinc finger motifs with two bound zinc atoms. The coiled-coil domain forms antiparallel dimers and serves as a platform for posttranslational modification and protein–protein interaction^5^. The C-terminal regions of TRIM proteins are quite divergent and often confer function specificity.

**Fig. 1:**
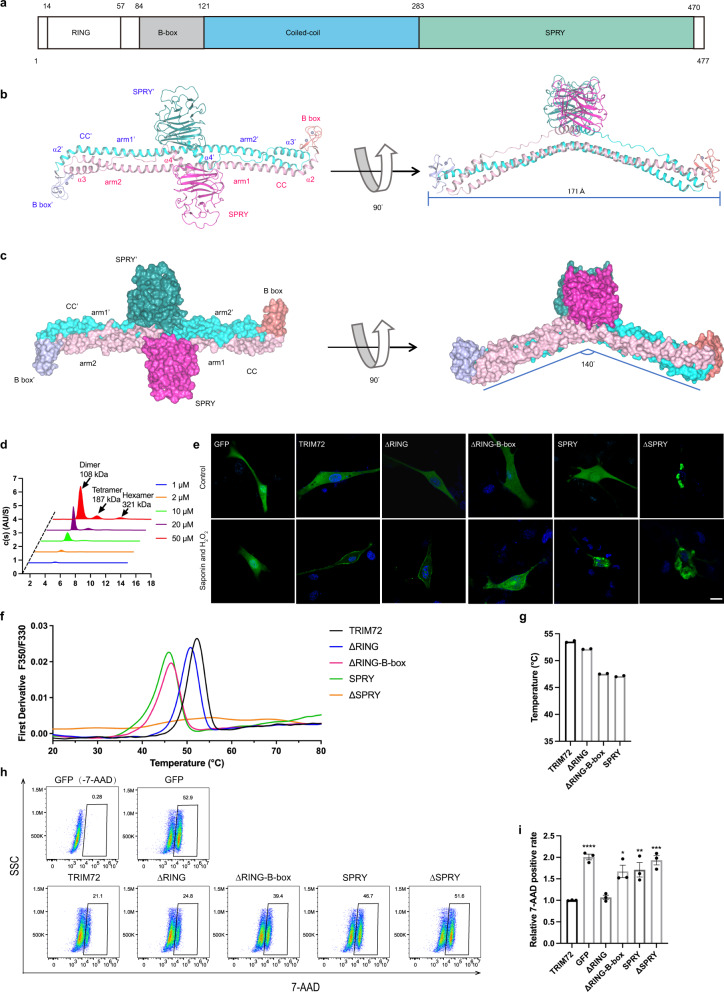
Overall structure of TRIM72 BCC-SPRY. **a** Domain architecture of TRIM72, the four domains are colored differently (RING in white, B-box in gray, coiled-coil in cyan, SPRY in green). **b** The overall structure of the TRIM72 BCC-SPRY homodimer in ribbon representation. The structure is displayed in two orientations, B-boxes are colored in light blue or salmon, coiled-coil domains in cyan or pink, and SPRY domains in teal or magenta. Symmetry mates are denoted by ′ next to the label. The α helices in the coiled-coil domain are as labeled as α2, α3, and α4. α1 is in B-box and not labeled. **c** Surface representation of TRIM72 BCC-SPRY homodimer, colored as in **b**. **d** SV-AUC analysis of TRIM72 at a range of protein concentrations: 1 μM (blue), 2 μM (orange), 10 μM (green), 20 μM (purple), and 50 μM (red). At 50 μM, a hexamer species appeared, accounting for 6.84% of the total protein. **e** Representative confocal images of C2C12 myoblasts expressing GFP, GFP-TRIM72, GFP-TRIM72^ΔRING^, GFP-TRIM72^ΔRING-B-box^, GFP-TRIM72^SPRY^, or GFP-TRIM72^ΔSPRY^ (green) with or without treatment with H_2_O_2_/saponin. The nucleus is stained with Hoechst (blue). TRIM72 (2-477), ∆RING (84-477), ∆RING-B-box (122-477), SPRY (278-477), ∆SPRY (2-287). Scale bar, 10 μm. Micrographs are representative of three independent experiments. **f**, **g** Thermal stabilities of TRIM72 deletion constructs measured by nano-DSF. The melting curves are shown in **f**, and temperatures of the Inflection Point for Ratio F350/F330 are shown in **g**. Data were from two independent experiments. Measurements of ∆SPRY (2-287) produced a very weak signal and were not used in **g**. **h** Cell viability analysis of HEK293T cells over-expressing various TRIM72 constructs after H_2_O_2_/saponin treatment. Cells were stained by 7-AAD and analyzed with flow cytometry; unstained cells were used for gating. Representative images of three independent experiments are shown. **i** Relative 7-AAD positive rate was calculated from **h** (*n* = 3, biological replicates). Data have been normalized to TRIM72 WT group; mean ± SEM; *p < 0.05, **p < 0.01, ***p < 0.001, ****p < 0.0001; one-way ANOVA with Dunnett΄s multiple comparison test. (GFP: *p* < 0.0001; ΔRING: *p* = 0.9996; ΔRING-B-box: *p* = 0.0129; SPRY: *p* = 0.0071; ΔSPRY: *p* = 0.0001). Source data are provided as a Source Data file.

Human TRIM72, a tripartite motif family protein primarily found in striated muscle, consists of four domains: the RING domain, B-box domain, coiled-coil domain, and SPRY domain (Fig. 1a and Supplementary Fig. 1). The RING domain is capable of E3 ubiquitin ligase activity and has been reported to be essential for TRIM72-induced degradation of the insulin receptor (IR), insulin receptor substrate 1 (IRS1), Focal adhesion kinase (FAK) and AMP-activated protein kinase α^6–9^. The B-box domain was shown to be able to mediate the interaction between TRIM72 and FAK^8^. Two leucine zipper motifs in the coiled-coil domain of TRIM72 (LZ1 and LZ2) are highly conserved in different animal species, and LZ1 is critical for forming TRIM72 dimer^10^. The SPRY region of TRIM72 has been demonstrated to interact with Orai1, enhancing extracellular Ca^2+^ entry into cells and reducing intracellular Ca^2+^ release through RyR1 to regulate skeletal muscle contraction^11^. TRIM72 could also bind to sarcoplasmic reticulum Ca^2+^-ATPase 1a (SERCA1a), reducing cytosolic Ca^2+^ levels to the resting level during skeletal muscle relaxation^12^. Since its identification, the TRIM72 protein has been associated with various physiological functions, including calcium homeostasis, muscle contraction, vesicle trafficking, membrane repair, anti-inflammation, reduction of oxidative stress, and regulation of systemic insulin response^13–18^. Among these, one key function of TRIM72 is membrane damage repair.

The integrity of the cell membrane is essential for maintaining cell morphology and protecting normal physiological activities. When a cell is exposed to external stimuli such as mechanical forces, chemical agents, drugs, or oxidative stress, it is critical that the intrinsic membrane repair mechanism is timely and effective to maintain the integrity of the cell membrane^19,20^. To reseal the membrane, three major steps must take place. First, the cell must be able to sense the destruction of the plasma membrane and where it is being destroyed. Second, intracellular vesicles need to be precisely transferred to the site of injury. Third, the vesicles fuse with the plasma membrane to repair the damage. TRIM72 can quickly sense the change of oxygen partial pressure after cell membrane damage, forming oligomers and carrying vesicles to complete the repair of the plasma membrane^18,21–23^. TRIM72 acts as a major regulator for membrane repair by interacting with dysferlin-1, caveolin-3 (Cav3), cavin-1, and Nonmuscle myosin type IIA (NM-IIA). Dysferlin, a protein involved in skeletal muscle membrane repair, is believed to fuse intracellular vesicles to repair damaged membranes^24^. GFP-dysferlin expressed in C2C12 myoblasts that do not express endogenous TRIM72 didn’t respond to cell damage because there was no recruitment of vesicles at the site of injury^25^. After co-expression of unlabeled TRIM72, GFP-dysferlin was transported to the site of plasma membrane injury. This result suggests that TRIM72 is required for the movement of dysferlin to sites of cell injury during repair patch formation^25^. Cav3 is a muscle-specific protein and its mutations are associated with muscular dystrophy. Co-expression studies showed that Cav3 could modulate the membrane damage repair ability of TRIM72. Cav3 P104L mutation severely attenuates the membrane repair function of TRIM72^21,25^. Polymerase I and transcript release factor (PTRF, also known as cavin-1), a protein known to regulate caveolae membrane structure, can recognize and bind the exposed cholesterol at the injury site and acts as a docking protein for TRIM72, allowing the formation of a membrane repair patch^26^. NM-IIA, a key cytoskeleton motor protein, interacts with TRIM72 to transport TRIM72-containing vesicles to the site of membrane injury^27^. Therefore, TRIM72 plays an indispensable role in membrane damage repair. *Trim72*^−/−^ mice display compromised sarcolemma repair with progressive myopathy^18^.

Besides striated muscle, TRIM72 also exists in human hearts and has protective roles for cardiac muscle injury since membrane rupture is a major cause of cardiomyocyte cell death^15,28^. Similarly, TRIM72 has been reported to be able to repair damaged lung epithelial cells and protect against acute kidney injury^29,30^. Moreover, as a muscle cytokine, TRIM72 can be secreted out of muscle cells to exert its protective effects^14^. In animal models, recombinant human TRIM72 has been demonstrated to protect against muscle dystrophy, acute myocardial, lung, and kidney injuries^23,29,31–34^. Due to its critical role in membrane repair, TRIM72 is emerging as a promising therapeutic target for the treatment of muscular dystrophy, cardiovascular, and kidney disease^23,28,29,31–37^.

Despite the extensive functional data on TRIM72, structural information is limited to the apo-structure of its SPRY domain^38^. To improve our understanding of the working mechanism of TRIM72, we have determined the crystal structure of the dimeric TRIM72 BCC-SPRY region. We also identified key amino acids affecting TRIM72 membrane localization and oligomerization through confocal microscopy and biochemical analysis. The effect of mutation of these residues was further assessed via cell viability assays. Our results provide a structural foundation for understanding TRIM72 action in membrane repair.

## Results

Purified full-length human TRIM72 protein (2-477) readily crystallizes under a few conditions. However, these crystals often diffract poorly, this might explain the lack of structural information for the full-length protein. After extensive optimization, we managed to obtain relatively well-diffracting crystals and determined the structure of the TRIM72 dimer at 3.0 Å (Fig. 1b, c, Supplementary Figs. 1, 2, and Supplementary Table 1). In the structure, we observed electron density for TRIM72 B-box, coiled-coil, and SPRY domains (BCC-SPRY), but the RING domain is not observed despite the fact that we used full-length protein for crystallization. Sequence analysis showed that the RING domain is tethered to the B-box by a flexible linker, and its position might be highly dynamic (Fig. 1a and Supplementary Fig. 1). The overall shape of TRIM72 BCC-SPRY dimer is similar to that of a clothes hanger (Fig. 1b, c). The coiled-coil region is the centerpiece of the protein and contains three α-helices (α2-α4) (Fig. 1b and Supplementary Fig. 1). Residues (122–230) form a long α2 helix which in turn forms an antiparallel dimer with the α2′ helix from the other molecule. The α3 helix containing residues 234–253 packs with the two α2 helices of the dimer to form a 3-helix bundle (Fig. 1b). A proline-rich loop connects α3 and α4 and lies on top of the α2 helix dimer. The short α4 helix (residues 270 to 283) again forms an antiparallel dimer with α4′ helix from the other protomer. Both ends of the coiled-coil domain are covered by a B-box domain. The two B-box and coiled-coil domains span a length of 171 Å with an angle of around 140° between its two arms. The two globular SPRY domains sit above the coiled-coil domain at the midpoint, with one on each side (Fig. 1b, c). Previous studies proposed that the dimeric TRIM72 could be further assembled into tetramer and high-order oligomers^10,18^. During protein purification, full-length TRIM72 protein was eluted on a Superdex 200 increase column with an apparent molecular weight of approximately 150 kDa (Supplementary Fig. 3a), which was larger than a dimer. We hypothesized that the higher apparent molecular weight of TRIM72 in gel filtration might be caused by its elongated shape. Sedimentation velocity analytical ultracentrifugation (SV-AUC) of TRIM72 in a buffer containing 0.3 mM TCEP showed that at lower concentrations, TRIM72 exists in solution as a dimer (Fig. 1d). With increasing protein concentration, tetramer and hexamer species appeared (Fig. 1d), the KD for tetramer formation was estimated to be around 780 μM based on methods reported by Zhao et al. (Supplementary Fig. 3b, c)^39^. Since the protein concentration used for measurement was not saturating, the high KD value is probably an approximate number, reflecting the weak association between TRIM72 dimers. TRIM72 displayed similar concentration-dependent weak oligomerization during SEC-MALS (size-exclusion chromatography coupled with multi-angle light scattering) analysis (Supplementary Fig. 3d, e). The observation of separated peaks in SV-AUC and SEC-MALS is likely due to the slow dissociation kinetics of TRIM72 dimer-tetramer equilibrium. In contrast, KAP1/TRIM28, which has a dimer-tetramer dissociation constant on the order of 10 μM, displayed a single peak with increasing molecular weights as the protein concentration increased, indicating rapidly interconverting dimer-tetramers^40^.

To probe the role of the different TRIM72 domains in membrane damage repair, we constructed different TRIM72 deletion mutants and used confocal microscopy to examine the functional effects of these mutations on TRIM72-mediated cell membrane repair in live C2C12 myoblast cells (Fig. 1e). C2C12 cells were reported to lack TRIM72 expression before differentiation^7,41,42^. A low concentration of saponin and H_2_O_2_ could partially penetrate the plasma membrane and cause membrane damage^43,44^. After cell injury, there was no change in GFP localization and no enrichment of GFP on the plasma membrane. Next, we detected GFP-labeled wild-type TRIM72 (GFP-TRIM72) localized to the cell membrane, intracellular vesicles, and cytoplasm. After cell injury, GFP-TRIM72 rapidly translocated to the plasma membrane in C2C12 myoblast cells (Fig. 1e and Supplementary Fig. 4a). The transfer of large amounts of GFP-TRIM72 to the cell membrane results in an obvious reduction of GFP-TRIM72 in the cytoplasm. However, most deletion constructs, with the exception of RING deletion (GFP-TRIM72^ΔRING^), accumulated in the cytoplasm and failed to translocate to the cell surface. Only a small fraction of GFP-TRIM72^ΔRING-B-box^ protein in the cytoplasm translocated to the membrane surface, indicating that deletion of B-box weakened the membrane damage repair ability of TRIM72 (Fig. 1e and Supplementary Fig. 4a). A large amount of GFP-TRIM72^ΔSPRY^ aggregated in the cytoplasm and couldn’t translocate to the membrane, indicating that deletion of the SPRY domain abolished the membrane repair ability. SPRY alone (GFP-TRIM72^SPRY^) was also unable to translocate to the cell surface (Fig. 1e). SV-AUC and SEC-MALS analysis showed that TRIM72^ΔRING^, TRIM72^ΔRING-B-box^, and TRIM72^ΔSPRY^ remained as a dimer in solution, SPRY alone is monomeric (Supplementary Fig. 5). Thermostability analysis by nano differential scanning fluorimetry (nano-DSF) revealed that WT TRIM72 and TRIM72^ΔRING^ have similar melting temperatures (Fig. 1f, g). TRIM72^ΔRING-B-box^ and SPRY both have reduced stability, which could partially explain their propensity to form aggregates in cells (Fig. 1e–g). We also tested the protective effect of TRIM72 against H_2_O_2_/saponin treatment in 293 T cells. Transfection with WT TRIM72 or TRIM72^ΔRING^ resulted in the lowest percentage of 7-AAD^+^ dead cells, while other constructs provided less protection (Fig. 1h, i). These results suggest that the RING motif of GFP-TRIM72 is probably not necessary for the repair of cell membranes, while TRIM72 B-box, coiled-coil, and SPRY domain all play critical roles in membrane damage repair.

**Fig. 2:**
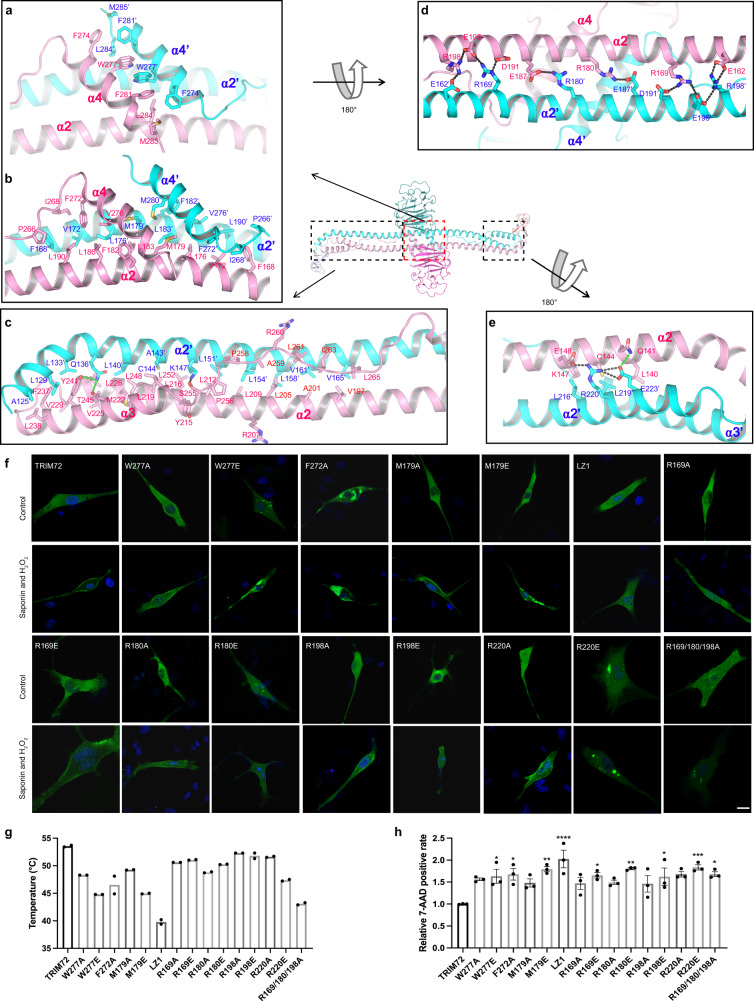
Structure of the coiled-coil domain. **a** Schematic showing the first layer of interacting hydrophobic residues between α4 helices, the two chains of TRIM72 dimer are colored pink and cyan, respectively. **b** Schematic showing the second and third layers of interacting residues at the α4-α2 junction. **c** Schematic showing the inner interface residues of the coiled-coil domain arm region. **d** Schematic showing the electrostatic network between α2-α2’, the view is rotated 180° from **a**. **e** Schematic showing the residues surrounding C144. **f** Representative confocal images of C2C12 myoblasts expressing GFP-TRIM72 mutants (W277A/E, F272A, M179A/E, LZ1, R169A/E, R180A/E, R198A/E, R220A/E, and R169/180/198 A) (green). The nucleus is stained with Hoechst (blue). Scale bar, 10 μm. Micrographs are representative of two independent experiments. **g** Thermal stabilities of TRIM72 coiled-coil domain mutants measured by nano-DSF. Temperatures of the Inflection Point for Ratio F350/F330 are shown; data were from two independent experiments. **h** The relative 7-AAD positive rate was calculated after cells expressing TRIM72 mutants were treated with H_2_O_2_/saponin (*n* = 3, biological replicates). Data have been normalized to TRIM72 WT group; mean ± SEM; *p < 0.05, **p < 0.01, ***p < 0.001, ****p < 0.0001; one-way ANOVA with Dunnett΄s multiple comparison test. (W277A: *p* = 0.0669; W277E: *p* = 0.0240; F272A: *p* = 0.0122; M179A: *p* = 0.1839; M179E: *p* = 0.0016; LZ1: *p* < 0.0001; R169A: *p* = 0.2049; R169E: *p* = 0.0176; R180A: *p* = 0.1580; R180E: *p* = 0.0013; R198A: *p* = 0.2300; R198E: *p* = 0.0279; R220A: *p* = 0.0767; R220E: *p* =  0.0008; and R169/180/198A: *p* = 0.0115). Source data are provided as a Source Data file.

In the TRIM72 structure, the α4 dimer packs tightly with the center region of the long α2 dimer to form an X-shaped junction with three layers of hydrophobic interactions (Fig. 2a, b). The top layer mainly consists of residues F274, W277, F281, L284, and M285 from α4 (Fig. 2a) and the middle layer is composed of F182, V276, M179, and M280 (Fig. 2b). The third layer involves a series of leucine residues (LZ1) from α2, namely L190, L186, L183, L176, and V172 (Fig. 2b). The loop region before α4 also contains a few hydrophobic residues (I268 and F272) that stabilize its interaction with α4 and α2 (Fig. 2b). Overall, the region around the α4-α2 junction appears to be quite rigid and the rigidity of this region might be critical for the proper function of TRIM72. To test this, we carried out point mutations (W277A or E, M179A or E, F272A) in the junction, aiming to disrupt the local interactions. SEC-MALS analysis of these mutant proteins showed that they remained dimeric (Supplementary Fig. 6a–f). However, in C2C12 cells, W277E, M179E, and F272A were prone to aggregation, especially after H_2_O_2_/saponin treatment (Fig. 2f), which is in agreement with their reduced thermal stability (Fig. 2g, Supplementary Fig. 8a). These mutants also had reduced ability to translocate to the membrane (Fig. 2f and Supplementary Fig. 4b). W277A or M179A mutations were less affected and had similar phenotype as WT (Fig. 2f and Supplementary Fig. 4b). The LZ1 region was previously shown to be essential for TRIM72 dimerization and membrane repair^10^. We mutated L190, L186, L183, L176, and M179 to alanine and found that the LZ1 mutant is mostly monomeric (Supplementary Fig. 6g). In C2C12 cells, LZ1 expressed normally but failed to translocate to the membrane after treatment with H_2_O_2_ and saponin (Fig. 2f and Supplementary Fig. 4b), in agreement with published results^10^. Not surprisingly, W277A or E, M179A or E, F272A, and LZ1 mutants offered less protection of cells from H_2_O_2_/saponin (Fig. 2h and Supplementary Fig. 9a). The data above suggested that the tightly formed junction of TRIM72 coiled-coil region plays critical roles for TRIM72 structural organization and membrane repair function.

The arm region of the coiled-coil domain, including the three-helix bundle at the tip, is stabilized by two groups of interacting residues (Fig. 2c). First, the three individual components of the arm are held together by a slew of hydrophobic residues at the inner core, including many leucine residues that were previously grouped as LZ2 (L205, L212, L219, and L226) (Fig. 2c)^10^. Second, the solvent-facing side of each α2 is lined with many charged residues, which form a series of electrostatic pairs (Fig. 2d, e). Worth mentioning is that E148-R220′-E223′-Q141 forms an electrostatic and hydrogen-bonding network that appears to bury C144 inside the arm (Fig. 2e). C144 was identified as a site for *S*-nitrosylation^43^. For *S*-nitrosylation to happen, R220′ needs to break away from interacting with E148 and E223′, which could serve as a regulation mechanism to control C144S-nitrosylation. To investigate the possible role of these charged residues in the arm region, we mutated some of the positively charged arginine residues (R169A or E, R180A or E, R198A or E, R220A or E) to see whether they had any effect on TRIM72 structure and function. In vitro purified mutant proteins remained in the dimer form (Supplementary Fig. 6h–p) and most of them displayed slightly reduced melting temperatures (Fig. 2g and Supplementary Fig. 8a). In C2C12 cells, these arginine mutations impaired TRIM72’s ability to translocate to the membrane to different degrees (Fig. 2f and Supplementary Fig. 4b). R220E and R169/180/198 A triple mutant, which are less stable, formed aggregates in the cells and failed to translocate to the membrane (Fig. 2f, g). In cell viability assays, all of the arginine mutations showed a reduced protection effect, but not as severe as the LZ1 mutation (Fig. 2h and Supplementary Fig. 9a). These data indicate that positively charged residues in the coiled-coil domain contribute to maintaining proper TRIM72 local structure and function. In addition, R207 and R260 residues which were reported to be involved in ADP-ribosylation^45^, reside at the middle part of the arm, and both are exposed to solvent, available for modification (Fig. 2c). Comparison of the coiled-coil domain of TRIM72 with those from other TRIM proteins with available structures^5,40,46–48^ showed that the clustered arginine residues are a unique feature of TRIM72 (Supplementary Fig. 10).

**Fig. 3:**
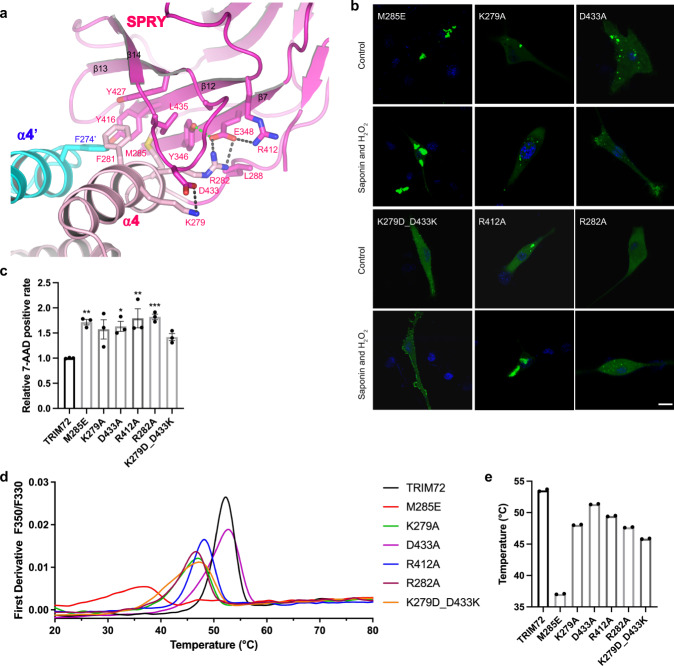
SPRY domain interacts with the coiled-coil domain. **a** Close-up view of the SPRY- α4 interface residues, green dashed lines indicate hydrogen bonds and black dash lines denote salt bridges. **b** Representative confocal images of C2C12 myoblasts expressing GFP-TRIM72 mutants (M285E, K279A, D433A, R412A, R282A, and K279D_D433K) with or without treatment with H_2_O_2_/saponin. The nucleus is stained with Hoechst (blue). Scale bar, 10 μm. Micrographs are representative of two independent experiments. **c** The relative 7-AAD positive rate was calculated after cells expressing TRIM72 mutants were treated with H_2_O_2_/saponin (*n* = 3, biological replicates). Data have been normalized to TRIM72 WT group; mean ± SEM; *p < 0.05, **p < 0.01, ***p < 0.001; one-way ANOVA with Dunnett΄s multiple comparison test. (M285E: *p* = 0.0063; K279A: *p* = 0.0563; D433A: *p* = 0.0243; R412A: *p* = 0.0016; R282A: *p* = 0.0010; K279D_D433K: *p* = 0.3443). **d**, **e** Thermal stabilities of TRIM72 mutants measured by nano-DSF. The melting curves are shown in **d**, and temperatures of the Inflection Point for Ratio F350/F330 are shown in **e**. Data were from two independent experiments. Source data are provided as a Source Data file.

The TRIM72 SPRY domain forms a highly twisted spherical β-sandwich structure consisting of 13 β-sheets and many connecting loops. The overall structure of the SPRY domain in TRIM72 is similar to the SPRY alone structure^38^, the superposition of two structures yielded an RMSD of 0.68 Å over 186 aligned Cα atoms (Supplementary Fig. 11). As shown in Fig. 3a, the SPRY domain is not flexibly linked to the coiled-coil domain as generally believed, instead, the SPRY domain of TRIM72 formed extensive interactions with the α4 helices. The interaction interface could be divided into two parts: on the left side, highly hydrophobic residues F274’, F281, Y416, Y427, L435, Y346, and M285 cluster together; on the right side, K279-D433, R282-E348-R412-Y346 interact through charges and hydrogen-bonding (Fig. 3a). Due to these interactions, SPRY is glued to the end of α4. Mutations of critical interface residues had little effect on the overall folding of purified proteins (Supplementary Fig. 7a–f) but led to aggregation and loss of TRIM72 repair ability to different extents in cells (Fig. 3b, c and Supplementary Figs. 4c, 9b). M285E, which presumably disrupted the hydrophobic interactions, caused GFP-TRIM72 to form large aggregates in C2C12 cells and the mutant protein failed to translocate to the plasma membrane after injury, which is also consistent with its significantly reduced melting temperature (Fig. 3b–e).

The positive charge of K279 was previously observed to be important, as K279R had no effect while other mutations impaired TRIM72 function^49^. Our structure provides a clear explanation for this observation: K279 stabilizes the loop between β13 and β14 by interacting with D433 (Fig. 3a). Indeed, in C2C12 cells, K279A or D433A formed aggregates, as shown by ref. ^49^. Interestingly, K279D D433K double mutation partially restored TRIM72’s ability to translocate to the plasma membrane in response to H_2_O_2_/saponin treatment (Fig. 3b and Supplementary Fig. 4c) and showed a better protection effect than K279A or D433A mutations in cell viability assays (Fig. 3c and Supplementary Fig. 9b). Taken together, these results pointed to the conclusion that SPRY must be firmly attached to the α4 and the correct conformation of SPRY-α4 interface is critical for TRIM72 function.

**Fig. 4:**
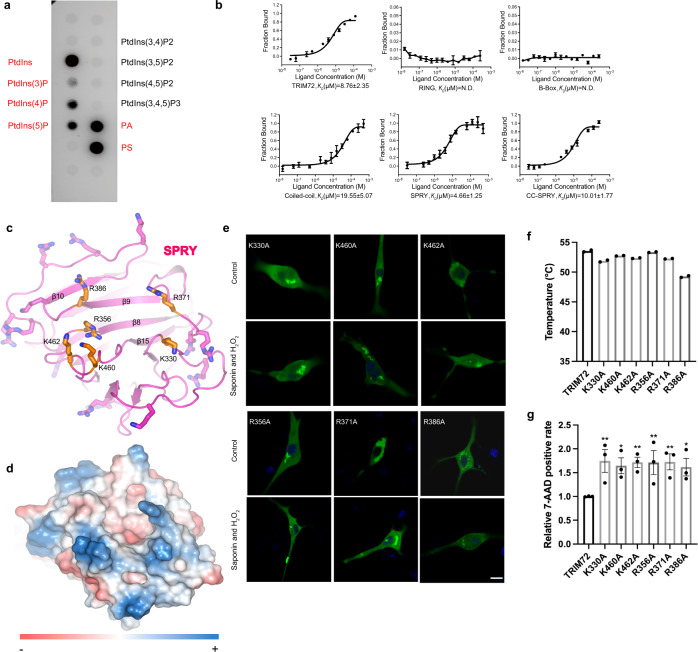
Analysis of TRIM72 lipid interaction. **a** PIP-Strips lipid dot blot analysis revealed that TRIM72 binds phosphatidylserine, phosphatidic acid, phosphatidylinositol (PtdIns), and phosphatidylinositol monophosphates, colored in red. **b** MST titration results of TRIM72 fragments with phospho-l-serine (PS). Data represent mean ± SEM from *n* = 3 independent experiments. **c** Schematic showing the positively charged lysine or arginine residues on the surface of SPRY domain, residues surrounding the cavity are highlighted with orange color. **d** The electrostatic potential surface of SPRY, positive and negative electrostatic potentials are colored blue and red, respectively. **e** Representative confocal images of C2C12 myoblasts expressing GFP-TRIM72 mutants (K330A, K460A, K462A, R356A, R371A, and R386A) with or without treatment with H_2_O_2_/saponin. The nucleus is stained with Hoechst (blue). Scale bar, 10 μm. Micrographs are representative of two independent experiments. **f** Thermal stabilities of SPRY mutants measured by nano-DSF. Temperatures of the Inflection Point for Ratio F350/F330 are shown; data were from two independent experiments. **g** The relative 7-AAD positive rate was calculated after cells expressing TRIM72 mutants were treated with H_2_O_2_/saponin (*n* = 3, biological replicates). Data have been normalized to TRIM72 WT group; mean ± SEM; *p < 0.05, **p < 0.01; one-way ANOVA with Dunnett΄s multiple comparison test. (K330A: *p*  = 0.0038; K460A: *p*  =  0.0201; K462A: *p* = 0.0064; R356A: *p* = 0.0069; R371A: *p* = 0.0055; R386A: *p* = 0.0310). Source data are provided as a Source Data file.

TRIM72 was previously shown to preferentially interact with phosphatidylserine, which is negatively charged^18,50^. Using PIP-Strips lipid dot blot analysis, we found that TRIM72 associated strongly with phosphatidylserine, phosphatidic acid, and phosphatidylinositol (PtdIns) (Fig. 4a). TRIM72 also displayed an affinity for phosphatidylinositol monophosphate. Results from microscale thermophoresis (MST) titration experiments confirmed that TRIM72 indeed interacts with these lipids’ headgroups (Fig. 4b and Supplementary Fig. 12a–c). We next tested the binding of different TRIM72 domains with phospho-L-serine (PS), the head group of phosphatidylserine. MST titration results showed that the RING and B-box domain have very low affinity for PS while the coiled-coil domain alone (121-277) has a modest affinity (KD ≈ 19.6 μM) (Fig. 4b). CC-SPRY and full-length TRIM72 have higher affinities for PS (KD ≈ 10 μM) (Fig. 4b). Mutation of the positively charged R169/180/198 residues in the coiled-coil domain reduced TRIM72’s affinity for PS by fourfold (KD ≈ 38 μM, Supplementary Fig. 12d), indicating these arginine residues might contribute to PS interaction. SPRY domain showed a relatively high affinity for PS (KD ≈ 4.7 μM, Fig. 4b). As shown in Fig. 1b, SPRY is globular and smaller in size, while TRIM72 constructs containing the coiled-coil domain are larger and elongated; it might be difficult to directly compare the MST affinity of SPRY alone toward PS with other TRIM72 constructs since measured fluorescence could be affected by factors such as size, charge and solvation entropy^51^. Nevertheless, it appears both the SPRY domain and the coiled-coil domain are able to interact with PS.

In the TRIM72 dimer, the two SPRY domains are quite close but not interacting with each other. One interesting feature is that the globular SPRY domain of 194 residues is lined with positively charged lysine and arginine residues (19 in total) on the surface (Fig. 4c, d). A crater-like cavity lies at the center of the SPRY domain, with three arginine and three lysine residues on the rim of the crater. To investigate the possible function of these residues, we generated six mutants (K330A, K460A, K462A, R356A, R371A, and R386A, Supplementary Fig. 7g–l). Among these mutations, R386A showed slightly reduced melting temperature and resulted in some aggregates in cells but was able to translocate to the membrane after stimulation with H_2_O_2_/saponin (Fig. 4e, f and Supplementary Figs. 8b, c, 4d); surprisingly all other five mutations aggregated in cells and lost the ability to translocate to the membrane in response to damage (Fig. 4e and Supplementary Fig. 4d). All six mutations exhibited less protection effect than WT with R386A performing slightly better than other mutations (Fig. 4g and Supplementary Fig. 9c). We also measured the binding affinity of the SPRY domain bearing these mutations toward PS (Supplementary Fig. 12e–j). SPRY^K460A^ and SPRY^K462A^ largely lost the ability to interact with PS; SPRY^R356A^, SPRY^R371A^, and SPRY^R386A^ showed reduced binding affinity, while SPRY^K330A^ was similar to SPRY^WT^. Thus, it appears K460, K462, and to some extent R356, R371A, and R386A are probably involved in interaction with PS while K330 is dispensable, indicting K330 may have certain other functions, considering its loss of function in cell-based assays. These results suggest that TRIM72 interaction with phosphatidylserine is mediated by SPRY in combination with the coiled-coil domain. As shown in Fig. 1e, a small amount of GFP-TRIM72^ΔRING-B-box^ is still translocated to the membrane after H_2_O_2_/saponin treatment. Both the SPRY and coiled-coil regions have many positively charged residues that could contribute to PS/lipid binding (Figs. 2c–e, 4c, d and Supplementary Fig. 10).

**Fig. 5:**
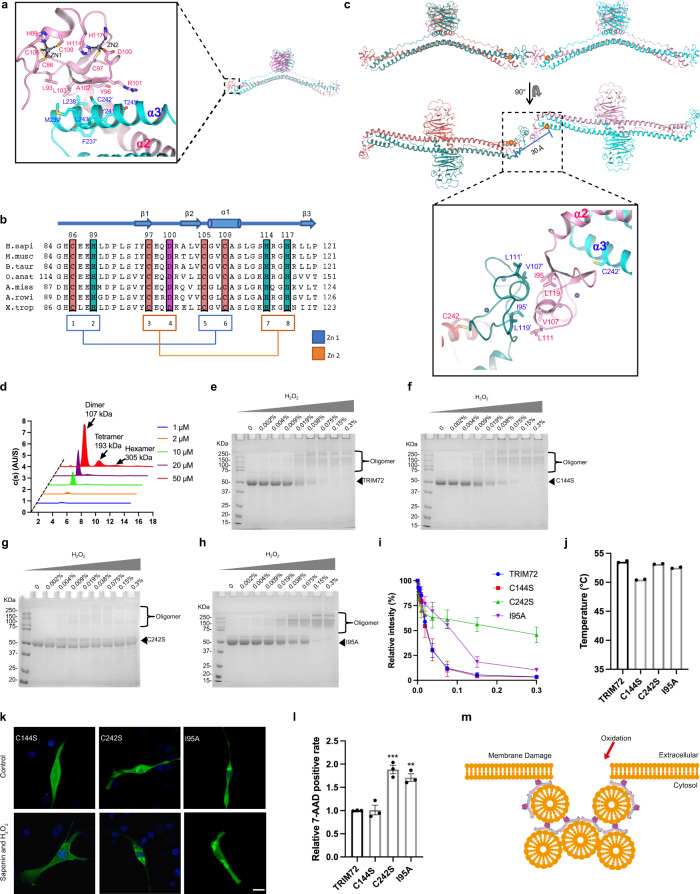
B-box dimerization promotes C242-mediated TRIM72 oligomerization. **a** Close-up view of TRIM72 B-box region. TRIM72 is colored as in Fig. 1. The zinc atoms (ZN1 and ZN2) are displayed as gray spheres. The residues coordinating ZN1 and ZN2 are shown as sticks. Residues that are involved in B-box, α3′ interaction are also shown. **b** Alignment of B-box domain sequences from several vertebrate species. Secondary structures of the B-box domain are drawn above the sequence alignment. The conserved Zn-binding residues are highlighted. **c** Ribbon representation of a TRIM72 tetramer mediated by B-box dimerization. B-box dimer interface residues (I95, V107, L111, and L119) are shown as sticks, and zinc atoms are displayed as gray spheres. Orange spheres denote C242 residues from two adjacent TRIM72 molecules. **d** SV-AUC analysis of TRIM72 I95A mutation at a range of protein concentrations: 1 μM (blue), 2 μM (orange), 10 μM (green), 20 μM (purple), and 50 μM (red). At 50 μM, the hexamer fraction is around 1.33%. **e**–**i** Non-reducing PAGE gel analysis of purified WT TRIM72, C144S, C242S, or I95A mutant proteins after incubation with different amounts of H_2_O_2_. The integrated intensities of the monomer band at 50 kDa were divided by the monomer band without H_2_O_2_ in each gel, and shown in **i**, data represent three independent experiments. Data represent mean ± SEM from *n* = 3 independent experiments. **j** Thermal stabilities of TRIM72 mutants measured by nano-DSF. Temperatures of the Inflection Point for Ratio F350/F330 are shown; data were from two independent experiments. **k** Representative confocal images of C2C12 myoblasts expressing GFP-TRIM72^C144S^ or GFP-TRIM72^C242S^ with or without treatment with H_2_O_2_/saponin. The nucleus is stained with Hoechst (blue). Scale bar, 10 μm. Micrographs are representative of two independent experiments. **l** The relative 7-AAD positive rate was calculated after cells expressing TRIM72 mutants were treated with H_2_O_2_/saponin (*n* = 3, biological replicates). Data have been normalized to TRIM72 WT group; mean ± SEM; *p < 0.05, **p < 0.01, ***p < 0.001; one-way ANOVA with Dunnett΄s multiple comparison test. (C144S: *p* > 0.9999; C242S: *p* = 0.0003; I95A: *p* = 0.0070). **m** A model of TRIM72 involvement in membrane repair. After plasma membrane damage, TRIM72 oligomerizes through oxidization-mediated C242 disulfide bond formation, which helps to nucleate vesicles and bring vesicles to the plasma membrane. The B-box domain promotes C242 disulfide bond formation through dimerization, while the coiled-coil and SPRY domains interact with vesicles and the plasma membrane. Source data are provided as a Source Data file.

The TRIM72 B-box domain is located at the end of the coiled-coil domain, acting like a cap (Figs. 1b, 5a). It adopts a typical B-box-type zinc finger fold with a conserved β1-β2-α-β3 arrangement (Fig. 5a, b). Zinc atom 1 (ZN1) is coordinated by C86, H89, C105, and C108; zinc atom 2 (ZN2) is coordinated by C97, D100, H114, and H117, the use of an aspartate instead of Cys/His for ZN2 coordination is similar to what has been observed for TRIM5α B2-box (Supplementary Fig. 13)^52^. Sequence alignment indicates that the residues that coordinate zinc atoms in TRIM72 B-box are highly conserved in vertebrates (Fig. 5b). The B-box is connected to the α2 helix via a short Pro-Ala-Ala linker and forms a nearly vertical turn (Fig. 5a and Supplementary Fig. 1). As a result, it actually sits on top of α3′ from the other protomer. Hydrophobic interactions mediated by residues from the B-box (L93, L103, A102, Y96, and the aliphatic portion of R101) and α3′ (M239, L238, and C242) might help to stabilize the B-box in the kinked orientation (Fig. 5a).

Cai et al. mutated 16 cysteine residues in mouse TRIM72 to alanine and showed that only the C242A mutation resulted in a complete loss of TRIM72 oligomerization, and it was demonstrated that C242 could mediate TRIM72 oligomerization by forming disulfide bonds under oxidative stress conditions^18^. Intriguingly, C242 is located at α3 and semi-covered by the B-box (Fig. 5a). What’s more, crystal packing analysis revealed that B-box is interacting with a nearby B-box from another molecule in the lattice (Fig. 5c). B-box is known to be able to form homo-oligomers^53–56^. One direct result of TRIM72 B-box dimerization is that the C242 residues from two TRIM72 molecules are brought to a close distance of 30 Å, possibly facilitating disulfide bond formation (Fig. 5c). The dimeric interface of B-box in the crystal is mediated by a few conserved hydrophobic residues, namely I95, L119, V107, and L111 (Fig. 5c).

SV-AUC showed that TRIM72 I95A also underwent concentration-dependent oligomerization (Fig. 5d and Supplementary Fig. 14a, b). Compared to WT, the proportion of oligomer at each concentration was slightly less (Supplementary Figs. 14a, 3b); more importantly, at 50 μM the I95A mutant produced much fewer hexamer species than WT (Figs. 1d, 5d). The KD for I95A tetramer formation was estimated to be around 2.2 mM (Supplementary Fig. 14b). I95A mutant also produced less tetramer fraction than WT in SEC-MALS analysis (Supplementary Figs. 14c, d, 3d, e). With H_2_O_2_ treatment and non-reducing SDS–PAGE gels, we showed that mutation of C242S but not C144S led to reduced TRIM72 oligomerization (Fig. 5e–g, i). I95A mutation also caused less TRIM72 oligomerization than WT (Fig. 5h, i), supporting B-box’s role in promoting TRIM72 association. SEC-MALS in the presence of H_2_O_2_ yielded similar results, with I95A forming fewer tetramers (Supplementary Fig. 15). Thermostability analysis by nano-DSF showed that these three mutations only had a minor reduction in their melting temperatures (Fig. 5j and Supplementary Fig. 8d). In cells, after H_2_O_2_/saponin treatment, GFP-TRIM72 C242S didn’t move to the membrane and lost the ability to respond to injury (Fig. 5k and Supplementary Fig. 4e), in agreement with previous findings^18,43^. C144S mutant acted similarly to WT (Fig. 5k and Supplementary Fig. 4e). As previously mentioned, C144 is buried by R220 and surrounding residues and blocked from forming disulfide bonds (Fig. 2e). I95A mutant formed aggregates in cells and couldn’t translocate to the membrane after damage (Fig. 5k and Supplementary Fig. 4e); I95A mutation also provided less protection from H_2_O_2_/saponin (Fig. 5l and Supplementary Fig. 9d). Together, these data demonstrated that B-box dimerization is critical for C242-mediated oligomerization of TRIM72 under oxidative stress conditions.

## Discussion

Plasma membrane integrity is vital for maintaining cellular homeostasis. The pioneering work by Cai et al. identified TRIM72/MG53 as an important player in acute membrane damage repair^18^. They also found that TRIM72 oligomerized in an oxidation-dependent manner through C242 and oligomerized TRIM72 could serve as a nucleation site to bring TRIM72-bound vesicles to the injury site and allow the formation of a repair complex^18^. A series of studies demonstrated the protective role of TRIM72 in multiple tissues, including muscle, heart, liver, kidney, and neurons^16,37,57–59^. Here through structural and biochemical characterization of the TRIM72 protein, we provided molecular insights into TRIM72’s working mechanism (Fig. 5m). The dimeric TRIM72 protein adopts an elongated shape with the coiled-coil domain forming its two arms, which would fit onto the surface of intracellular vesicles (Fig. 5m). The interaction could be mediated by the two arginine clusters in the coiled-coil domain (Fig. 2c–e and Supplementary Fig. 10), as arginine is able to form extensive electrostatic and hydrogen-bonding interactions and is believed to play a more prominent role than lysine when interacting with membrane lipids^60^. The B-box is at the end of each arm, and dimerization of the B-box could lead to the formation of linear TRIM72 oligomers (Fig. 1d). However, the interaction between B-boxes is likely to be dynamic as there is only a small fraction of TRIM72 exist as tetramers (Fig. 1d). It’s also possible that the weak B-box interaction in solution could be strengthened once TRIM72 is membrane-bound, as often seen for membrane-associated proteins, when their movement is limited to 2D instead of 3D. B-box dimerization greatly enhances C242-C242 disulfide bond formation under oxidative conditions (Fig. 5i and Supplementary Fig. 15e). Certain conformation changes of the B-box must take place during this process to reduce the distance between C242s from 30 Å to less than 3 Å. As shown by the recently reported 3.5 Å TRIM72 cryo-EM structure, the B-box region is highly dynamic and couldn’t be resolved in the cryo-EM map^61^. Molecular dynamics simulation of TRIM72 BCC-SPRY crystal structure showed that rotation of the B-box relative to the coiled-coil domain occurred without disintegrating the ternary structure of individual domains, which led to the exposure of C242 to the solvent (Supplementary Fig. 16, Supplementary Movie 1, and Supplementary Data 1, 2). These evidences suggest that the dynamics of the B-box could serve as a regulation mechanism for C242 disulfide bond formation, exposing C242 only when necessary. B-box dimerization seems to be a reasonable way to induce conformational changes of the B-box to open up while keeping two C242 residues close. What we captured in the crystal packing might be a snapshot of TRIM72 before C242 forms intermolecular disulfide bonds. Disulfide bond formation/oligomerization would bring TRIM72-containing vesicles to the plasma membrane or other TRIM72-containing vesicles to carry out membrane patch repair. The rigidly associated SPRY domain has lipid binding capacity (Figs. 3a, 4b–d), and it might mediate vesicle-vesicle, vesicle-plasma membrane fusion (Fig. 5m), besides its role in protein–protein interaction.

In addition to the linear mode of oligomerization, it’s possible that TRIM72 could pack in a side-by-side manner to produce a mesh network (Supplementary Fig. 17a, b). The side-by-side oligomerization was previously proposed for TRIM20 (PDB:4CG4), TRIM20 B30.2 (SPRY) domain interacts with the proline-rich loop from a nearby symmetry molecule (Supplementary Fig. 17c)^46^. In the TRIM72 crystal lattice, the TRIM72 SPRY domain also packs with a symmetry mate and contacts the three-helix bundle region of the coiled-coil domain (Supplementary Fig. 17a, b). However, we didn’t observe specific contacts between residues. So it remains to be investigated whether/how TRIM72 forms non-linear high-order assembly. The RING domain, which was missing in our crystal structure due to flexibility, might also contribute to TRIM72 oligomerization, similar to RING domains in other TRIM proteins^62–66^. Deletion of the RING domain reduced TRIM72 tetramer fraction during SV-AUC (Supplementary Fig. 5h, i); more studies would be required to reveal its precise role in TRIM72 oligomerization. At the moment, it seems the RING domain and its associated E3 ligase activity is dispensable for H_2_O_2_/saponin-induced membrane damage repair as TRIM72^ΔRING^ produced similar phenotypes as full-length protein in cell-based assays (Fig. 1e, i).

Interestingly, with lipid dot blot analysis, we found that TRIM72 interacted with several kinds of negatively charged lipids but was not limited to phosphatidylserine (Fig. 4a and Supplementary Fig. 12a–c), this is different from the results obtained by ref. ^18^. What’s more, TRIM72 displayed a preference for PtdIns and PtdIns mono phosphate but not PtdIns di- or tri-phosphate (Fig. 4a). The specificity and binding mechanism of TRIM72 towards lipids would require further investigation.

Our current model is limited to the actions of TRIM72 alone; many other protein factors like caveolin, dysferlin, PTRF, and calcium ion also play important roles in the membrane repair process. Moreover, posttranslational modifications like ADP-ribosylation and S-nitrosylation intricately modulate TRIM72 functions^43,45^. Further studies would be needed to elucidate how TRIM72 works synergistically with different partners as well as regulatory factors.

## Methods

### Cloning of human TRIM72

Total RNA was extracted using TriZol reagent according to the manual provided by the company (Life Technologies Co). The cDNA was generated using the cDNA Synthesis Kit Manual (Takara Bio) and the open reading frame of TRIM72 (GenBank: NM_001008274.4) was amplified by PCR. The PCR reactions were done using 2 × Phanta® Max Master Mix (Vazyme Biotech Co) according to the following program: 98 °C for 3 min (once), followed by 30 cycles (98 °C for 15 s, 58 °C for 15 s, and 72 °C for 50 s ), followed by 72 °C for 5 min. The DNA fragment was inserted into the pMD®18-T easy vector using TA cloning. The gel extraction kit and plasmid DNA miniprep kit was purchased from AXYGEN. The sequences were confirmed by DNA sequencing.

### Protein expression and purification

TRIM72 DNA sequences were inserted into a modified pRSFDuet-1 vector (Novagen) with an N-terminal His-Sumo tag using ClonExpress® II one-step Cloning Kit (Vazyme Biotech Co). The point mutations were carried out using the Fast Mutagenesis System (TransGen Biotech). The primers used are listed in Supplementary Data 3. The recombinant plasmids were transformed into *Escherichia coli* Rosetta (DE3) cells (Novagen) for protein expression. Bacteria cells were cultured for 3 to 4 h at 37 °C. After OD600 was about 0.6, 0.5 mM IPTG was added into the culture to induce expression of the recombinant proteins at 18 °C for 16 h. The cells were harvested by centrifugation (2348×*g*, 6 min), resuspended in a buffer containing (20 mM Tris-HCl, 200 mM NaCl, 0.3 mM TCEP, pH 8.0), and lysed using a cell disruptor (JNBIO). Cell debris was removed by centrifugation and the lysate was loaded onto a pre-equilibrated nickel Sepharose column (His Trap HP, GE Healthcare). The column was washed with buffer containing (20 mM Tris-HCl, 20 mM Imidazole, 200 mM NaCl, 0.3 mM TCEP, pH 8.0) and TRIM72 was eluted with a buffer containing (20 mM Tris-HCl, 250 mM Imidazole, 200 mM NaCl, 0.3 mM TCEP, pH 8.0). The His-sumo tag was cleaved by Ulp1 at 4 °C overnight during dialysis. The cleaved His-sumo tag and un-cleaved TRIM72 were removed by recycling over a pre-equilibrated nickel column. TRIM72 was purified further on a Source Q column (Cytiva) and a Superdex 200 increase gel filtration column (Cytiva) in a buffer containing 20 mM Tris-HCl, 200 mM NaCl, 0.3 mM TCEP, pH 8.0. The purity was checked by SDS–PAGE.

### Crystallization and data collection

Crystals of TRIM72 were grown in 24-well plates using the hanging drop vapor diffusion method by mixing 1.2 μL protein (15 mg/mL) with 1.2 μL crystallization buffer containing 100 mM HEPES pH 7.5, 11% (w/v) PEG 8000, 5% methanol. Crystal grew to full size after incubation at 16 °C for 2 weeks. Harvested crystals were stabilized in the well solution plus 30% glycerol and flash-frozen with liquid nitrogen. Diffraction data were collected at Beamline station BL19U1 at Shanghai Synchrotron Radiation Facility (SSRF, Shanghai, China).

### Structure determination and refinement

The data were integrated and scaled using XDS, the CCP4 program Pointless and Aimless^67–69^. The structure of TRIM72 was determined by molecular replacement using the SPRY structure from PDB 3KB5 as an initial search model with Phaser^70^. The structural model was built using Coot^71^ and refined using PHENIX^72^. Figures were generated using PyMOL (The PyMOL Molecular Graphics System, Version 2.3.4 Schrödinger, LLC). The statistics of the data collection and refinement are shown in Supplementary Table 1.

### Cell culture and transfection

The fragments used for expressing GFP-TRIM72 constructs were inserted into the plasmid pcDNA3.1(+). Point mutations in TRIM72 were carried out using the Fast Mutagenesis System (TransGen Biotech). C2C12 murine myoblast cells (SCSP-505, Cell bank of the Chinese Academy of Sciences) were grown in a humidified environment at 37 °C and 5% CO_2_ in Dulbecco’s modified Eagle’s medium, supplemented with 10% fetal bovine serum, 100 units/ml penicillin, and 100 μg/ml streptomycin. C2C12 cells were seeded into glass-bottom dishes (Corning, NY, USA) at 2 × 10^6^ cells and cultured overnight. The next day, 2 µg of each plasmid and 8 µL of Lipo3000 transfection reagent (Promega Co. Madison, WI, USA) were mixed and transferred to each well for transfection.

### Confocal imaging

After transfection, cells were incubated for 24 h, then the nucleus was stained with Hoechst 33342 Staining Solution for Live Cells (Beyotime) for 10 min, imaged under a Nikon C2 confocal microscope. GFP fusion proteins were excited with blue light and the nucleus stained with Hoechst 33342 was excited with UV light. The images of cells excited with individual fluorescent channels were taken separately and merged afterward with Nikon imaging software.

### Cell membrane damage assay

C2C12 cells were seeded into glass-bottom ΔT dishes at 2 × 10^6^ cells and grown overnight. Afterward, they were transfected as described above and incubated for 24 h. After washing cells once using Tyrode solution (140 mM NaCl, 2.5 mM CaCl_2_, 5 mM KCl, 10 mM HEPES, 2 mM MgCl_2_, pH 7.2), cells were immediately treated with Tyrode solution containing 0.005% saponin and 0.003% H_2_O_2_ for 5 min. All images were captured on a Nikon C2 confocal microscope as described above. Fluorescence intensity before and after H_2_O_2_/saponin treatment was analyzed by the Volocity software (v6.1.1). The ratio F1/F0 represents the change of fluorescence intensity on the cell membrane, where F0 is the average fluorescence intensity of three points in the cytoplasm (areas with protein aggregations were avoided) and F1 is the average fluorescence intensity of three points on the cell membrane. Intensity changes of five cells from each group were used for data analysis.

### Cell viability assay

HEK293T cells (CRL-3216, ATCC) were plated on 24‐well plates with a density of 10^5^ cells/ml for cell viability assay. After 12 h of culture, the cells were transfected as described above and incubated for 24 h. The cells were treated with Tyrode solution containing 0.005% saponin and 0.003% H_2_O_2_. Each treatment was in triplicate, and three independent experiments were performed. The cells were then prepared for viability assessment using a 7‐AAD kit (Yeasen Biotechnology) according to the manufacturer’s protocol, and analyzed on a CytoFlex flow cytometer (Beckman Coulter). Gating for 7-AAD positive cells was based on a control sample without 7-AAD. The data were analyzed using FlowJo (v9.3.1) software.

### In vitro H_2_O_2_ oxidation assay

The oxidation assay was performed with purified TRIM72 and mutant proteins in gel filtration buffer containing 20 mM Tris-HCl, pH 8.0, 200 mM NaCl, and 0.3 mM TCEP. About 20 μM TRIM72 was incubated with an increasing concentration of H_2_O_2_ (0–0.3%) at 4 °C for 30 min. TRIM72 oligomerization was visualized by SDS–PAGE. The results from three repeated experiments were analyzed using GraphPad Prism software (v9.0).

### PIP-strips assay

Membrane lipid binding analysis of FLAG-tagged TRIM72 was conducted using PIP-Strips^TM^ membranes (Thermo Fisher), with each nitrocellulose membrane containing 100 pmol of 15 different phospholipids and a blank sample. The membrane was blocked by TBST with 3% fatty acid-free BSA for 1 h at room temperature, followed by incubation with 5 μg/mL TRIM72-FLAG protein in TBST with 3% fatty acid-free BSA at 4 °C overnight. After washing three times with TBST, the membrane was blotted with anti-Flag Rabbit monoclonal antibody (1:1000, Cell Signaling Technology, cat#14793 S) overnight at 4 °C. After washing three times with TBST, the membrane was then incubated with HRP-labeled Goat Anti-Rabbit IgG (H + L) (1:5000, Beyotime Biotechnology, cat#A0208) for 1 h and detected with a chemiluminescence imaging system (Clinx Science).

### SEC-MALS analysis

Size-exclusion chromatography was performed with inline multi-angle laser light scattering using a Wyatt HELEOS-II 18-angle photometer coupled to a Wyatt Optilab rEX differential refractometer (Wyatt Technology Corp). About 100 μL protein at a concentration of 1 mg/mL in 20 mM Tris-HCl pH 8.0, 200 mM NaCl, 0.3 mM TCEP were applied onto a Superdex 200 increase column (GE Healthcare) at a flow rate of 0.5 mL/min. The data were analyzed using ASTRA (v6.1).

### SV-AUC analysis

Purified TRIM72 and mutants were diluted in 20 mM Tris-HCl pH 8.0, 200 mM NaCl, and 0.3 mM TCEP. Samples (400 μL) were loaded into 12-mm centerpieces and centrifuged at 141,995×*g* at 20 °C with an An-50Ti rotor in an Optima XL-I analytical ultracentrifuge (Beckman). The partial specific volume of different protein samples and the buffer density were calculated using the program SEDNTERP (http://www.rasmb.org/sednterp/). The final sedimentation velocity data were analyzed and fitted to a continuous sedimentation coefficient distribution model using the program SEDFIT (sedfitsedphat.nibib.nih.gov/software). The program SEDPHAT was used to perform quantitative analysis on the SV data for a self-association system and determine the corresponding dissociation constant (KD).

### Nano-DSF measurement

A tryptophan-fluorescence-based thermal unfolding experiment was performed using Prometheus NT.48 nano-DSF (Nanotemper Technologies). The capillaries containing 10 μL protein (20 μM) were inserted into the machine, the temperature was increased at a rate of 1 °C/min from 20 °C to 95 °C, and the fluorescence intensities at emission wavelengths of 330 and 350 nm were measured. The ratio of fluorescence intensities at 350 and 330 nm was used as a function of temperature to determine the protein inflection point, which is the temperature at which half of the protein is unfolded.

### MST titration

GFP-labeled protein samples and ligands were diluted with MST buffer (50 mM Tris pH 8.0, 80 mM NaCl, 0.05% TWEEN 20, 0.3 mM TCEP) and loaded into the capillaries of Monolith NT.115 (Nanotemper Technologies). For PS (phospho-l-serine) binding experiments, the protein target was fixed at 200 nM, and PS was titrated from 500 μM to 0.15 nM. After 15 min of incubation at room temperature, samples were loaded into Monolith NT.115 capillaries, and the data were analyzed using the MO. Affinity Analysis software (v2.3) provided by the manufacturer (NanoTemper).

### Steered molecular dynamics simulations

TRIM72 dimer was placed in a water box with a size of 11.7 × 11.3 × 20.2 nm^3^. TIP3P water and 150 mM NaCl were added to the box to mimic the physiological environment. The simulation system included 260,765 atoms. The CHARMM36 force field^73,74^ was used to describe the protein. The simulation was carried out with the GROMACS 2022.3 package (www.gromacs.org). An energy minimization with the combined steepest descent and conjugate gradient methods was first applied to the system. Afterward, the system was gradually heated to 310 K within 100 ps and held in NVT ensemble for 100 ps and then NPT ensemble for 1 ns with the protein constrained. The temperature and pressure were controlled by V-rescale^75^ and Parrinello–Rahman algorithms^76,77^, respectively. Afterward, the 20 ns MD simulations without any constraints were applied to the protein. The final configuration obtained by MD simulations was used as the initial model for steered molecular dynamics (sMD) simulations. In the sMD simulation, two B-boxes were pulled in opposite directions away from each other. All the Cα atoms in the B-box were subject to the force through a spring of 50 kJ/mol/nm moving at the speed of 0.5 nm/ns for 20 ns. The package Visual Molecular Dynamics (VMD 1.9.3)^78^ was used to visualize the simulation results. The SASA was calculated with VMD built-in tools.

### Statistical analysis

All statistical analyses were performed using GraphPad Prism (v9.0) Software. The data from all experiments were presented as mean ± SEM. Statistical analyses were performed with one‐way ANOVA followed by Dunnett’s test for three or more groups of data with multiple comparisons.

### Reporting summary

Further information on research design is available in the Nature Portfolio Reporting Summary linked to this article.

## Supplementary information

Supplementary information

Description of Additional Supplementary Files

Supplementary Data 1

Supplementary Data 2

Supplementary Data 3

Supplementary Movie 1

Reporting Summary

## Data Availability

The data that support this study are available from the corresponding authors upon request. Coordinates and structure factors are available in the Protein Data Bank (PDB) with accession code 7XT2 (TRIM72). Other PDB entries used in this study: 4CG4 (TRIM20), 3KB5 (TRIM72 SPRY), 6QAJ (TRIM28), 4NQJ (TRIM69), 4LTB (TRIM25), 5W9A (TRIM5α), and 5EIU (TRIM5α B-Box). Source data are provided as a Source Data file. Source data are provided with this paper.

## Source data

Source Data

## References

[1] Hatakeyama S (2017). TRIM family proteins: roles in autophagy, immunity, and carcinogenesis. Trends Biochem. Sci..

[2] van Tol, S., Hage, A., Giraldo, M. I., Bharaj, P. & Rajsbaum, R. The TRIMendous role of TRIMs in virus-host interactions. *Vaccines*10.3390/vaccines5030023 (2017). 10.3390/vaccines5030023 PMC5620554 28829373

[3] van Gent M, Sparrer KMJ, Gack MU (2018). TRIM proteins and their roles in antiviral host defenses. Annu. Rev. Virol..

[4] Esposito D, Koliopoulos MG, Rittinger K (2017). Structural determinants of TRIM protein function. Biochem. Soc. Trans..

[5] Sanchez JG (2014). The tripartite motif coiled-coil is an elongated antiparallel hairpin dimer. Proc. Natl Acad. Sci. USA.

[6] Song R (2013). Central role of E3 ubiquitin ligase MG53 in insulin resistance and metabolic disorders. Nature.

[7] Yi JS (2013). MG53-induced IRS-1 ubiquitination negatively regulates skeletal myogenesis and insulin signalling. Nat. Commun..

[8] Nguyen N, Yi JS, Park H, Lee JS, Ko YG (2014). Mitsugumin 53 (MG53) ligase ubiquitinates focal adhesion kinase during skeletal myogenesis. J. Biol. Chem..

[9] Jiang P (2021). Negative regulation of AMPK signaling by high glucose via E3 ubiquitin ligase MG53. Mol. Cell.

[10] Hwang M, Ko JK, Weisleder N, Takeshima H, Ma J (2011). Redox-dependent oligomerization through a leucine zipper motif is essential for MG53-mediated cell membrane repair. Am. J. Physiol. Cell Physiol.

[11] Ahn MK (2016). Mitsugumin 53 regulates extracellular Ca(2+) entry and intracellular Ca(2+) release via Orai1 and RyR1 in skeletal muscle. Sci. Rep..

[12] Lee KJ (2012). Mitsugumin 53 attenuates the activity of sarcoplasmic reticulum Ca(2+)-ATPase 1a (SERCA1a) in skeletal muscle. Biochem. Biophys. Res. Commun..

[13] Benissan-Messan DZ (2020). Multi-cellular functions of MG53 in muscle calcium signaling and regeneration. Front. Physiol..

[14] Whitson BA, Tan T, Gong N, Zhu H, Ma J (2021). Muscle multiorgan crosstalk with MG53 as a myokine for tissue repair and regeneration. Curr. Opin. Pharmacol..

[15] Zhong W, Benissan-Messan DZ, Ma J, Cai C, Lee PHU (2021). Cardiac effects and clinical applications of MG53. Cell Biosci..

[16] Zhang Y, Wu HK, Lv F, Xiao RP (2017). MG53: biological function and potential as a therapeutic target. Mol. Pharmacol..

[17] Wu HK (2019). Glucose-sensitive myokine/cardiokine MG53 regulates systemic insulin response and metabolic homeostasis. Circulation.

[18] Cai C (2009). MG53 nucleates assembly of cell membrane repair machinery. Nat. Cell Biol..

[19] McNeil PL, Ito S (1989). Gastrointestinal cell plasma membrane wounding and resealing in vivo. Gastroenterology.

[20] McNeil PL, Steinhardt RA (2003). Plasma membrane disruption: repair, prevention, adaptation. Annu. Rev. Cell Dev. Biol..

[21] Cai C (2009). MG53 regulates membrane budding and exocytosis in muscle cells. J. Biol. Chem..

[22] McNeil P (2009). Membrane repair redux: redox of MG53. Nat. Cell Biol..

[23] Weisleder N (2012). Recombinant MG53 protein modulates therapeutic cell membrane repair in treatment of muscular dystrophy. Sci. Transl. Med..

[24] Han R, Campbell KP (2007). Dysferlin and muscle membrane repair. Curr. Opin. Cell Biol..

[25] Cai C (2009). Membrane repair defects in muscular dystrophy are linked to altered interaction between MG53, caveolin-3, and dysferlin. J. Biol. Chem..

[26] Zhu H (2011). Polymerase transcriptase release factor (PTRF) anchors MG53 protein to cell injury site for initiation of membrane repair. J. Biol. Chem..

[27] Lin P (2012). Nonmuscle myosin IIA facilitates vesicle trafficking for MG53-mediated cell membrane repair. FASEB J..

[28] Wang X (2010). Cardioprotection of ischemia/reperfusion injury by cholesterol-dependent MG53-mediated membrane repair. Circ. Res..

[29] Duann P (2015). MG53-mediated cell membrane repair protects against acute kidney injury. Sci. Transl. Med..

[30] Nagre N (2016). TRIM72 modulates caveolar endocytosis in repair of lung cells. Am. J. Physiol. Lung Cell Mol. Physiol..

[31] Liu J (2015). Cardioprotection of recombinant human MG53 protein in a porcine model of ischemia and reperfusion injury. J. Mol. Cell Cardiol..

[32] Jia Y (2014). Treatment of acute lung injury by targeting MG53-mediated cell membrane repair. Nat. Commun..

[33] Kim SC (2014). TRIM72 is required for effective repair of alveolar epithelial cell wounding. Am. J. Physiol. Lung Cell Mol. Physiol..

[34] Liu C (2020). MG53 protects against contrast-induced acute kidney injury by reducing cell membrane damage and apoptosis. Acta Pharmacol. Sin..

[35] Lemckert FA (2016). Lack of MG53 in human heart precludes utility as a biomarker of myocardial injury or endogenous cardioprotective factor. Cardiovasc. Res..

[36] Shan D (2020). Cardiac ischemic preconditioning promotes MG53 secretion through H2O2-activated protein kinase C-delta signaling. Circulation.

[37] Li, Z. et al. MG53, a tissue repair protein with broad applications in regenerative medicine. *Cells*10.3390/cells10010122 (2021). 10.3390/cells10010122 PMC7827922 33440658

[38] Park EY (2010). Crystal structure of PRY-SPRY domain of human TRIM72. Proteins.

[39] Zhao H (2020). Quantitative analysis of protein self-association by sedimentation velocity. Curr. Protoc. Protein Sci..

[40] Stoll GA (2019). Structure of KAP1 tripartite motif identifies molecular interfaces required for retroelement silencing. Proc. Natl Acad. Sci. USA.

[41] Jung SY, Ko YG (2010). TRIM72, a novel negative feedback regulator of myogenesis, is transcriptionally activated by the synergism of MyoD (or myogenin) and MEF2. Biochem. Biophys. Res. Commun..

[42] Lee CS (2010). TRIM72 negatively regulates myogenesis via targeting insulin receptor substrate-1. Cell Death Differ..

[43] Kohr MJ, Evangelista AM, Ferlito M, Steenbergen C, Murphy E (2014). S-nitrosylation of TRIM72 at cysteine 144 is critical for protection against oxidation-induced protein degradation and cell death. J. Mol. Cell Cardiol..

[44] Weisleder, N. et al. Visualization of MG53-mediated cell membrane repair using in vivo and in vitro systems. *J. Vis. Exp.*10.3791/2717 (2011). 10.3791/2717 PMC3197044 21750489

[45] Ishiwata-Endo, H. et al. Role of a TRIM72 ADP-ribosylation cycle in myocardial injury and membrane repair. *JCI Insight*10.1172/jci.insight.97898 (2018). 10.1172/jci.insight.97898 PMC6302937 30429362

[46] Weinert C, Morger D, Djekic A, Grutter MG, Mittl PR (2015). Crystal structure of TRIM20 C-terminal coiled-coil/B30.2 fragment: implications for the recognition of higher order oligomers. Sci. Rep..

[47] Li Y (2014). Structural insights into the TRIM family of ubiquitin E3 ligases. Cell Res.

[48] Keown JR (2018). A helical LC3-interacting region mediates the interaction between the retroviral restriction factor Trim5alpha and mammalian autophagy-related ATG8 proteins. J. Biol. Chem..

[49] Zhou L, Middel V, Reischl M, Strahle U, Nienhaus GU (2018). Distinct amino acid motifs carrying multiple positive charges regulate membrane targeting of dysferlin and MG53. PLoS ONE.

[50] Kim S, Seo J, Ko YG, Huh YD, Park H (2012). Lipid-binding properties of TRIM72. BMB Rep..

[51] Sparks, R. P. & Fratti, R. Use of Microscale Thermophoresis (MST) to Measure Binding Affinities of Components of the Fusion Machinery. *Methods Mol. Biol.* **1860**, 191–198 (2019). 10.1007/978-1-4939-8760-3_11 PMC8466250 30317505

[52] Keown JR, Goldstone DC (2016). Crystal structure of the Trim5alpha Bbox2 domain from rhesus macaques describes a plastic oligomerisation interface. J. Struct. Biol..

[53] Li Y (2019). B1 oligomerization regulates PML nuclear body biogenesis and leukemogenesis. Nat. Commun..

[54] Diaz-Griffero F (2009). A B-box 2 surface patch important for TRIM5alpha self-association, capsid binding avidity, and retrovirus restriction. J. Virol..

[55] Huang SY (2014). The B-box 1 dimer of human promyelocytic leukemia protein. J. Biomol. NMR.

[56] Wagner, J. M. et al. Mechanism of B-box 2 domain-mediated higher-order assembly of the retroviral restriction factor TRIM5alpha. *Elife*10.7554/eLife.16309 (2016). 10.7554/eLife.16309 PMC4936894 27253059

[57] Blazek AD, Paleo BJ, Weisleder N (2015). Plasma membrane repair: a central process for maintaining cellular homeostasis. Physiology.

[58] Yao Y (2016). MG53 permeates through blood-brain barrier to protect ischemic brain injury. Oncotarget.

[59] Han, Y. et al. Membrane-delimited signaling and cytosolic action of MG53 preserve hepatocyte integrity during drug-induced liver injury. *J. Hepatol*. 10.1016/j.jhep.2021.10.017 (2021). 10.1016/j.jhep.2021.10.017 34736969

[60] Li L, Vorobyov I, Allen TW (2013). The different interactions of lysine and arginine side chains with lipid membranes. J. Phys. Chem. B.

[61] Niu Y (2022). Cryo-EM structure of human MG53 homodimer. Biochem. J..

[62] Koliopoulos MG, Esposito D, Christodoulou E, Taylor IA, Rittinger K (2016). Functional role of TRIM E3 ligase oligomerization and regulation of catalytic activity. EMBO J.

[63] Anandapadamanaban M (2019). E3 ubiquitin-protein ligase TRIM21-mediated lysine capture by UBE2E1 reveals substrate-targeting mode of a ubiquitin-conjugating E2. J. Biol. Chem..

[64] Wang P (2018). RING tetramerization is required for nuclear body biogenesis and PML sumoylation. Nat. Commun..

[65] Kiss L (2019). A tri-ionic anchor mechanism drives Ube2N-specific recruitment and K63-chain ubiquitination in TRIM ligases. Nat. Commun..

[66] Dawidziak DM, Sanchez JG, Wagner JM, Ganser-Pornillos BK, Pornillos O (2017). Structure and catalytic activation of the TRIM23 RING E3 ubiquitin ligase. Proteins.

[67] Evans PR, Murshudov GN (2013). How good are my data and what is the resolution?. Acta Crystallogr. D Biol. Crystallogr..

[68] Kabsch W (2010). Xds. Acta Crystallogr. D Biol. Crystallogr..

[69] Winn MD (2011). Overview of the CCP4 suite and current developments. Acta Crystallogr. D Biol. Crystallogr..

[70] McCoy AJ (2007). Phaser crystallographic software. J. Appl. Crystallogr..

[71] Emsley P, Cowtan K (2004). Coot: model-building tools for molecular graphics. Acta Crystallogr. D Biol. Crystallogr..

[72] Liebschner D (2019). Macromolecular structure determination using X-rays, neutrons and electrons: recent developments in Phenix. Acta Crystallogr. D Struct. Biol..

[73] MacKerell AD (1998). All-atom empirical potential for molecular modeling and dynamics studies of proteins. J. Phys. Chem. B.

[74] Huang J, MacKerell AD (2013). CHARMM36 all-atom additive protein force field: validation based on comparison to NMR data. J. Comput. Chem..

[75] Bussi G, Donadio D, Parrinello M (2007). Canonical sampling through velocity rescaling. J. Chem. Phys..

[76] Parrinello M, Rahman A (1981). Polymorphic transitions in single crystals: a new molecular dynamics method. J. Appl. Phys..

[77] Nosé S, Klein ML (2006). Constant pressure molecular dynamics for molecular systems. Mol. Phys..

[78] Humphrey W, Dalke A, Schulten K (1996). VMD: visual molecular dynamics. J. Mol. Graph..

