# Supplementary material for: Structural basis for TRIM72 oligomerization during membrane damage repair
Source: Nat Commun. 2023 Mar 21;14:1555. doi: 10.1038/s41467-023-37198-1 (PMC10030467; doi:10.1038/s41467-023-37198-1)
Supplement: Supplementary file 1 — Supplementary information [file 41467_2023_37198_MOESM1_ESM.pdf]

SUPPLEMENTARY INFORMATION

Structural basis for TRIM72 oligomerization during membrane damage repair

Yuemin Ma[1#], Lei Ding[1#], Zhenhai Li[2] and Chun Zhou[1]*

[1] School of Public Health, and Sir Run Run Shaw Hospital, Zhejiang University School of Medicine, Hangzhou, Zhejiang 310058, China.

2 Shanghai Key Laboratory of Mechanics in Energy Engineering, Shanghai Institute of Applied Mathematics and Mechanics, School of Mechanics and Engineering Science, Shanghai University, Shanghai 200072, China

[#] These authors contributed equally.

[*] Correspondence: chunzhou@zju.edu.cn

Supplementary Figures

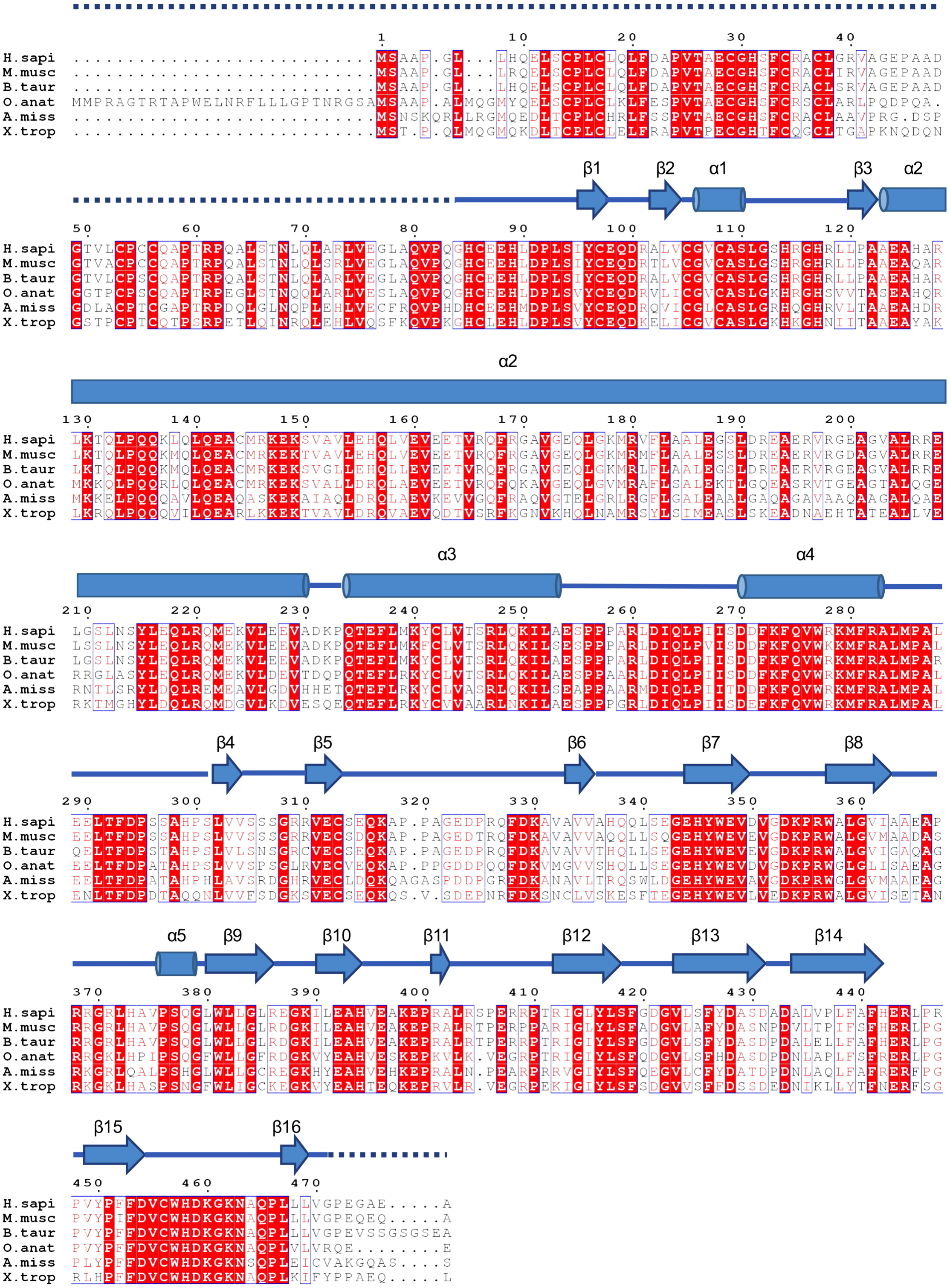

Supplementary Fig. 1

Sequence alignment of the TRIM72 from several vertebrates. The secondary structure is drawn above the sequences. Identically conserved residues are shaded red.

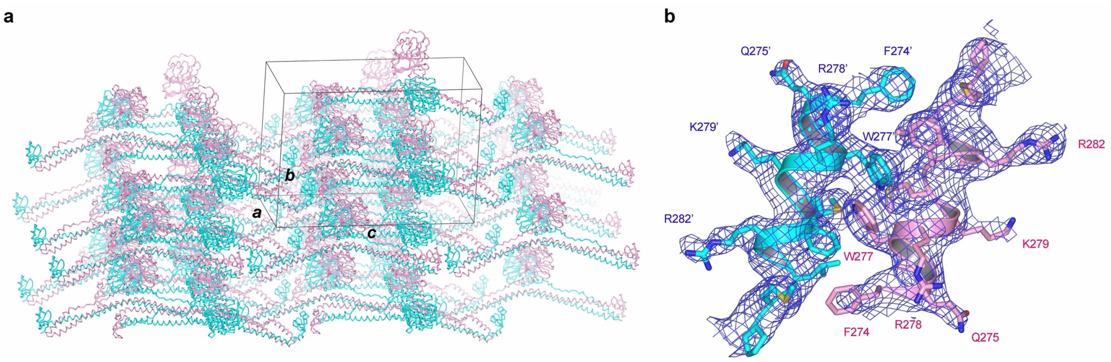

Supplementary Fig. 2

(**a**) An example of the crystal lattice, with the asymmetric unit indicated in black, the two molecules of TRIM72 dimer are colored in cyan and pink, respectively.

(**b**) Composite omit map (blue mesh) of TRIM72 α4 region contoured at 1.0 σ showing the electron density of side chains.

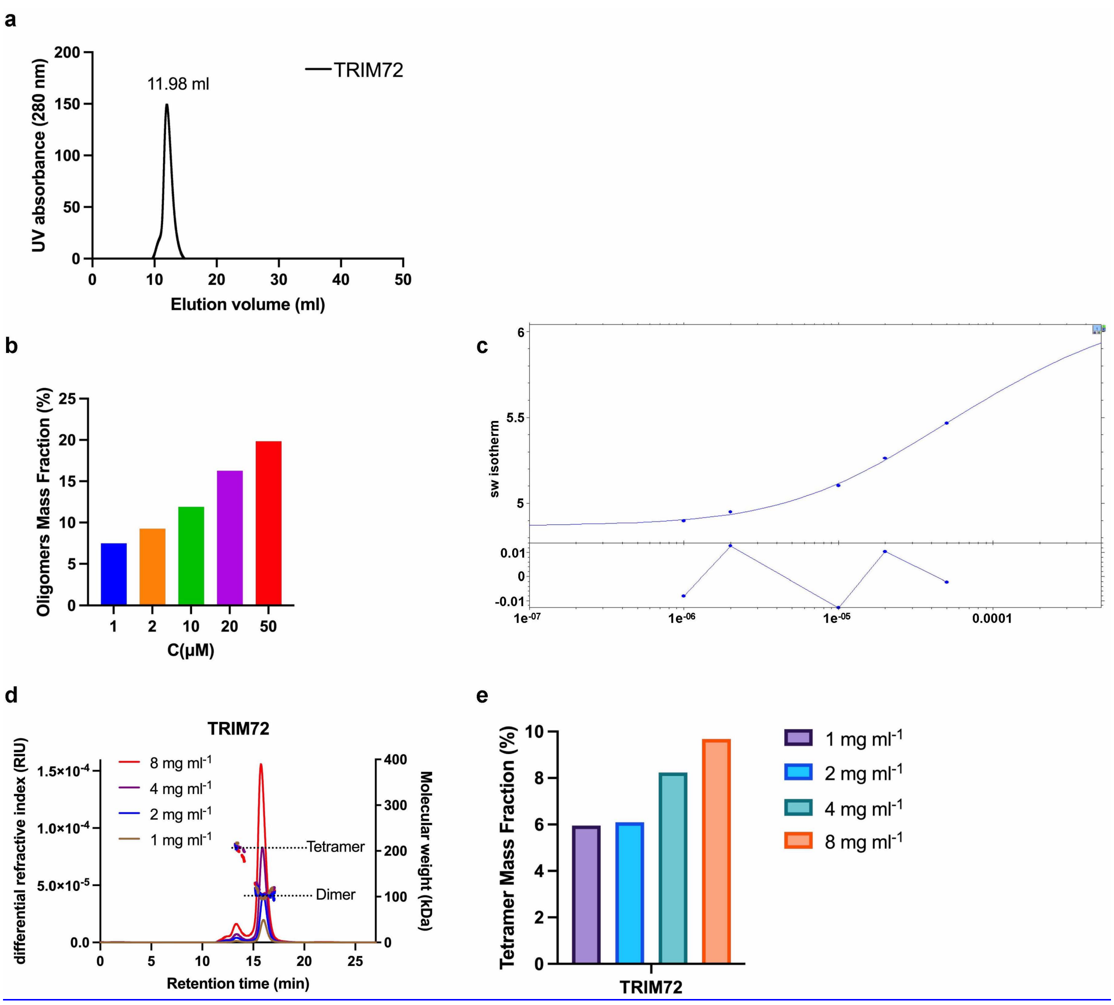

Supplementary Fig. 3

(**a**) The elution profile of TRIM72 on a Superdex 200 increase column.

(**b**-**c**) The percentage of oligomer mass fraction of TRIM72 in SV-AUC at various protein concentrations. Signal weighted-average sedimentation coefficient (sw) isotherm analysis in SEDPHAT is shown in **c**, the KD value for tetramer formation is calculated to be around 780 μM.

(**d**-**e**) SEC-MALS analysis of TRIM72 at a range of protein concentrations. The percentage of tetramer mass fraction of TRIM72 is shown in **e**.

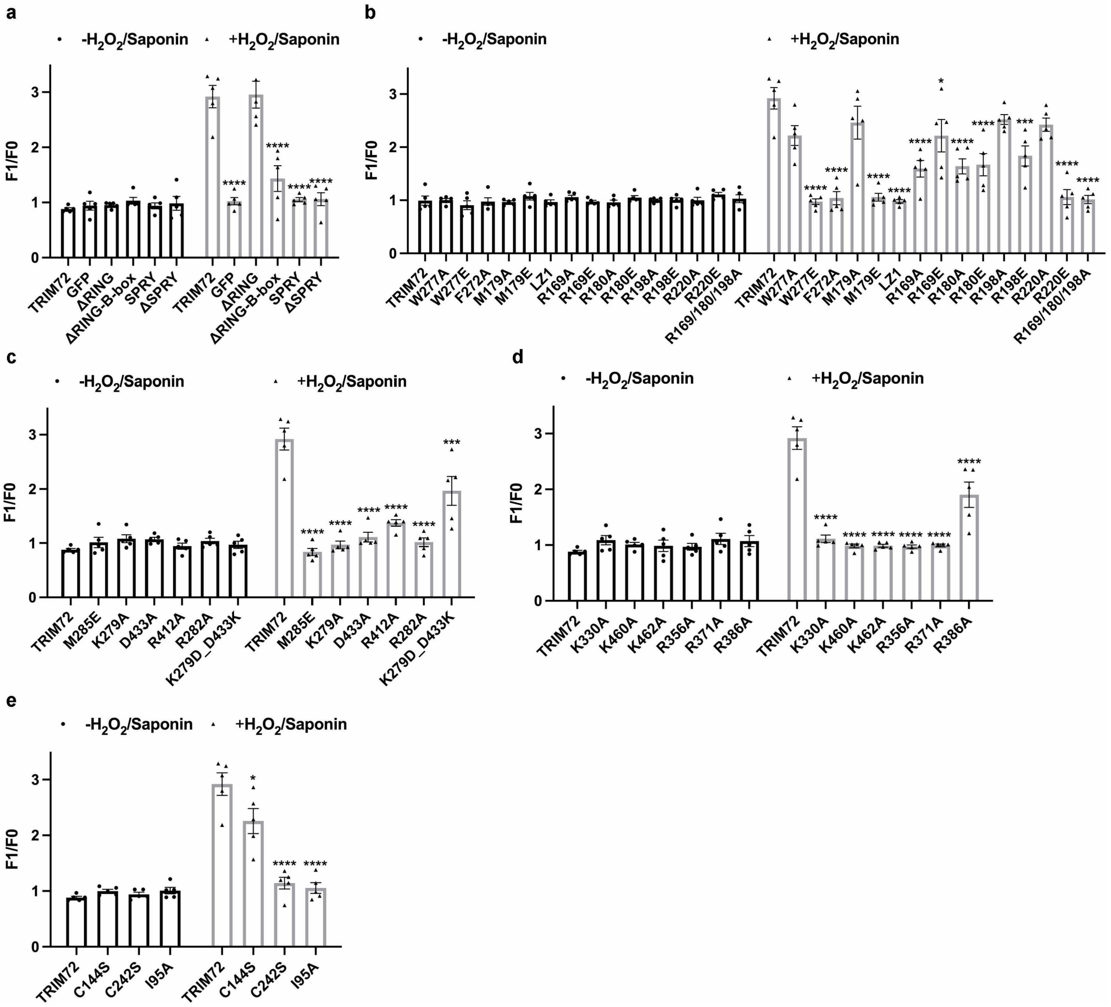

Supplementary Fig. 4

(a-e) Quantification of TRIM72 membrane translocation before and after H_2O_2/saponin treatment, related to Fig. 1e, 2f, 3b, 4e, 5k. The ratio F1/F0 represents the change of fluorescence intensity on the cell membrane, where F0 is the average fluorescence intensity of three points in the cell, and F1 is the average fluorescence intensity of three points on the cell membrane. Data are from five cells for each group; mean ± SEM, *p < 0.05, **p < 0.01, ***p < 0.001, ****p < 0.0001; one-way ANOVA with Dunnett´s multiple comparison test. -H_2O_2/saponin: **a** (GFP: p = 0.9594; ΔRING: p = 0.9262; ΔRING-B-box: p = 0.4304; SPRY: p = 0.9661; ΔSPRY: p = 0.7624); **b** (W277A: p = 0.9999; W277E: p = 0.9162; F272A: p = 0.9997; M179A: p = 0.9995; M179E: p = 0.9375; LZ1: p = 0.9996; R169A: p = 0.9879; R169E: p = 0.9997; R180A: p = 0.9994; R180E: p = 0.9952; R198A: p = 0.9998; R198E: p = 0.9998; R220A: p > 0.9999; R220E: p = 0.6587; and R169/180/198A: p = 0.9993); **c** (M285E: p = 0.4497; K279A: p = 0.1225; D433A: p = 0.1517; R412A: p = 0.9461; R282A: p = 0.2979; K279D_D433K: p = 0.8002); **d** (K330A: p = 0.2801; K460A: p = 0.7180; K462A: p = 0.8550; R356A: p = 0.9235; R371A: p = 0.1990; R386A: p = 0.3598); **e** (C144S: p = 0.1390; C242S: p = 0.6227; I95A: p = 0.1071). +H_2O_2/saponin: **a** (GFP: p < 0.0001; ΔRING:

$p = 0.9998$; ΔRING-B-box: $p < 0.0001$; SPRY: $p < 0.0001$; ΔSPRY: $p < 0.0001$); **b** (W277A: $p = 0.0511$; W277E: $p < 0.0001$; F272A: $p < 0.0001$; M179A: $p = 0.4146$; M179E: $p < 0.0001$; LZ1: $p < 0.0001$; R169A: $p < 0.0001$; R169E: $p = 0.0494$; R180A: $p < 0.0001$; R180E: $p < 0.0001$; R198A: $p = 0.5915$; R198E: $p = 0.0004$; R220A: $p = 0.3160$; R220E: $p < 0.0001$; and R169/180/198A: $p < 0.0001$); **c** (M285E: $p < 0.0001$; K279A: $p < 0.0001$; D433A: $p < 0.0001$; R412A: $p < 0.0001$; R282A: $p < 0.0001$; K279D_D433K: $p = 0.0003$); **d** (K330A: $p < 0.0001$; K460A: $p < 0.0001$; K462A: $p < 0.0001$; R356A: $p < 0.0001$; R371A: $p < 0.0001$; R386A: $p < 0.0001$); **e** (C144S: $p = 0.0327$; C242S: $p < 0.0001$; I95A: $p < 0.0001$).

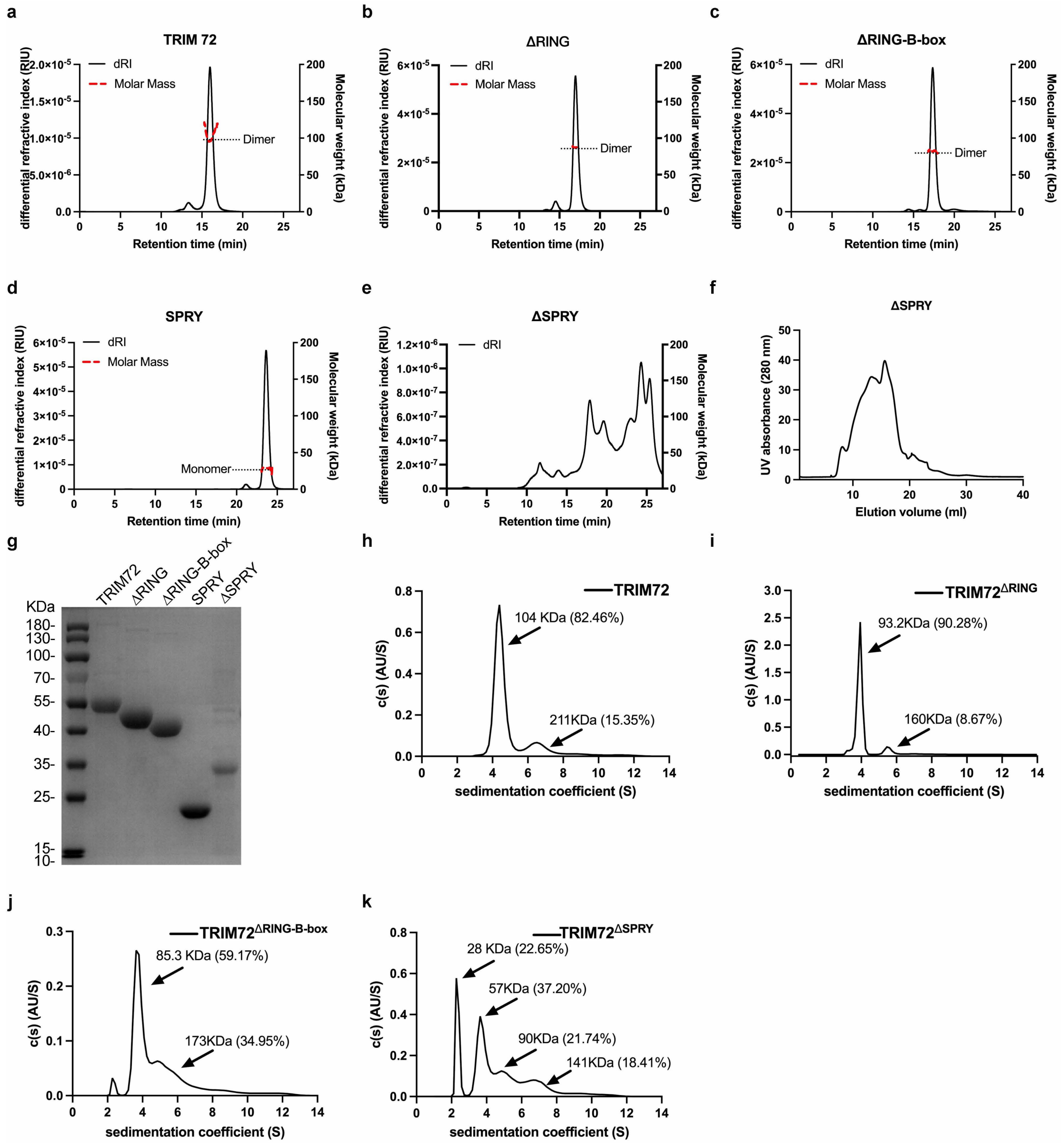

Supplementary Fig. 5

(**a**-**e**) SEC-MALS results of TRIM72 WT and truncation constructs, protein concentration is 1 mg/mL.

(**f**) The elution profile of TRIM72$^{\Delta SPRY}$ on a Superdex 200 increase column.

(**g**) SDS-PAGE gel of purified TRIM72 proteins, each protein sample was purified and examined by SDS-PAGE gels separately at least twice.

(**h**-**k**) SV-AUC results for TRIM72 WT and truncation constructs, protein concentration is at 1 mg/mL.

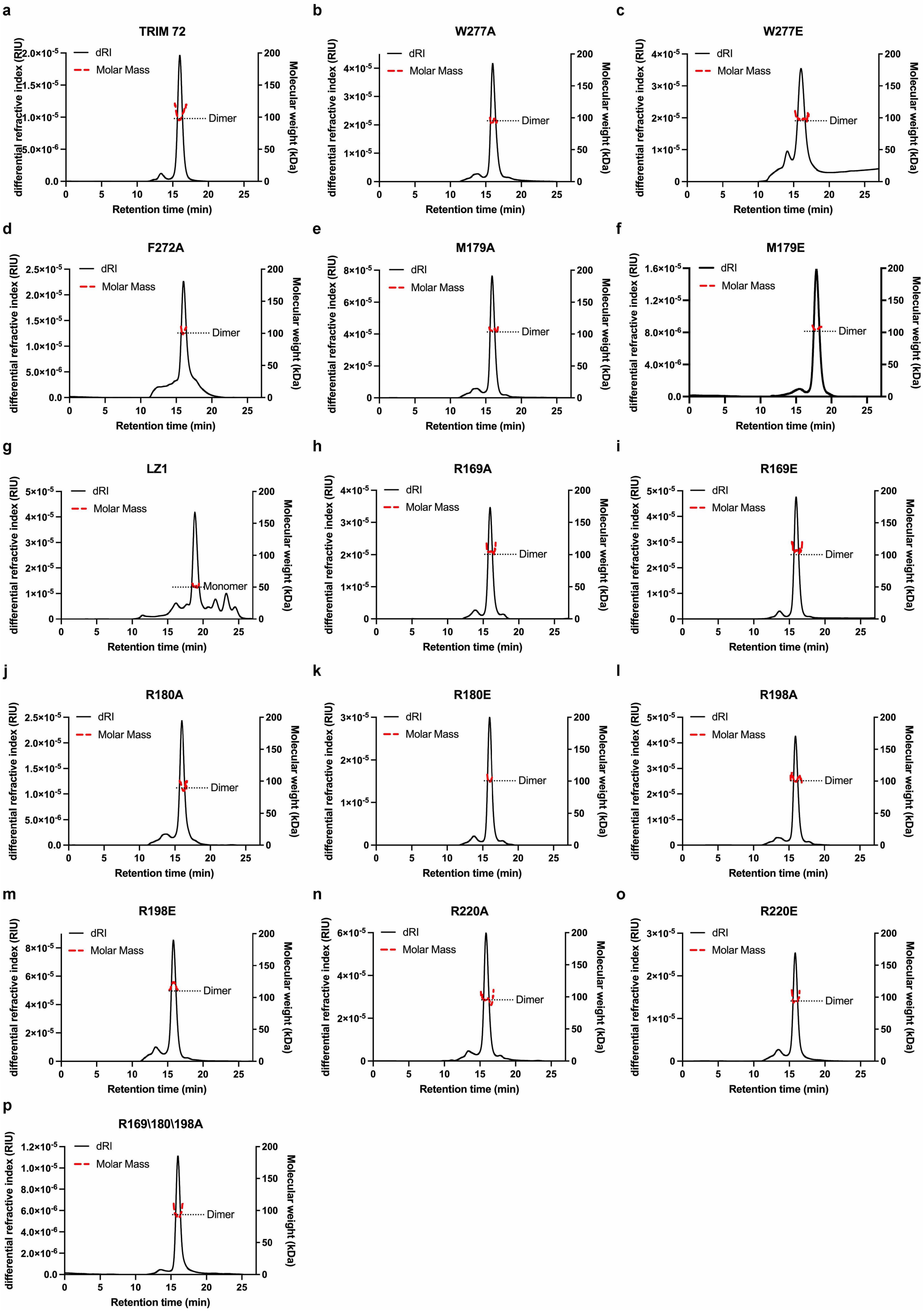

Supplementary Fig. 6

(**a-p**) SEC-MALS results of TRIM72 WT and mutant proteins, protein concentration is 1 m g/mL.

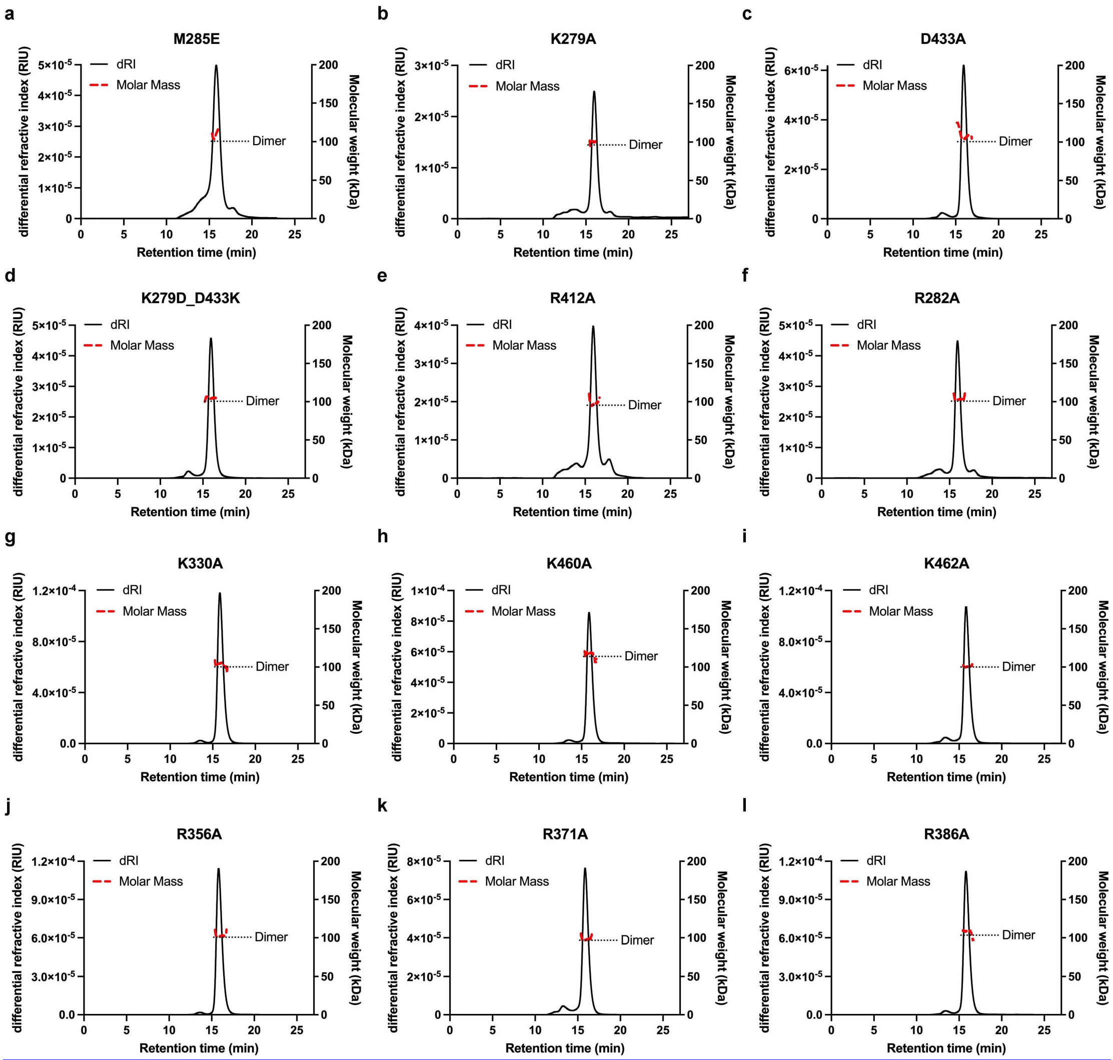

Supplementary Fig. 7

(a-l)SEC-MALS results of TRIM72 mutant proteins, protein concentration is 1 mg /mL.

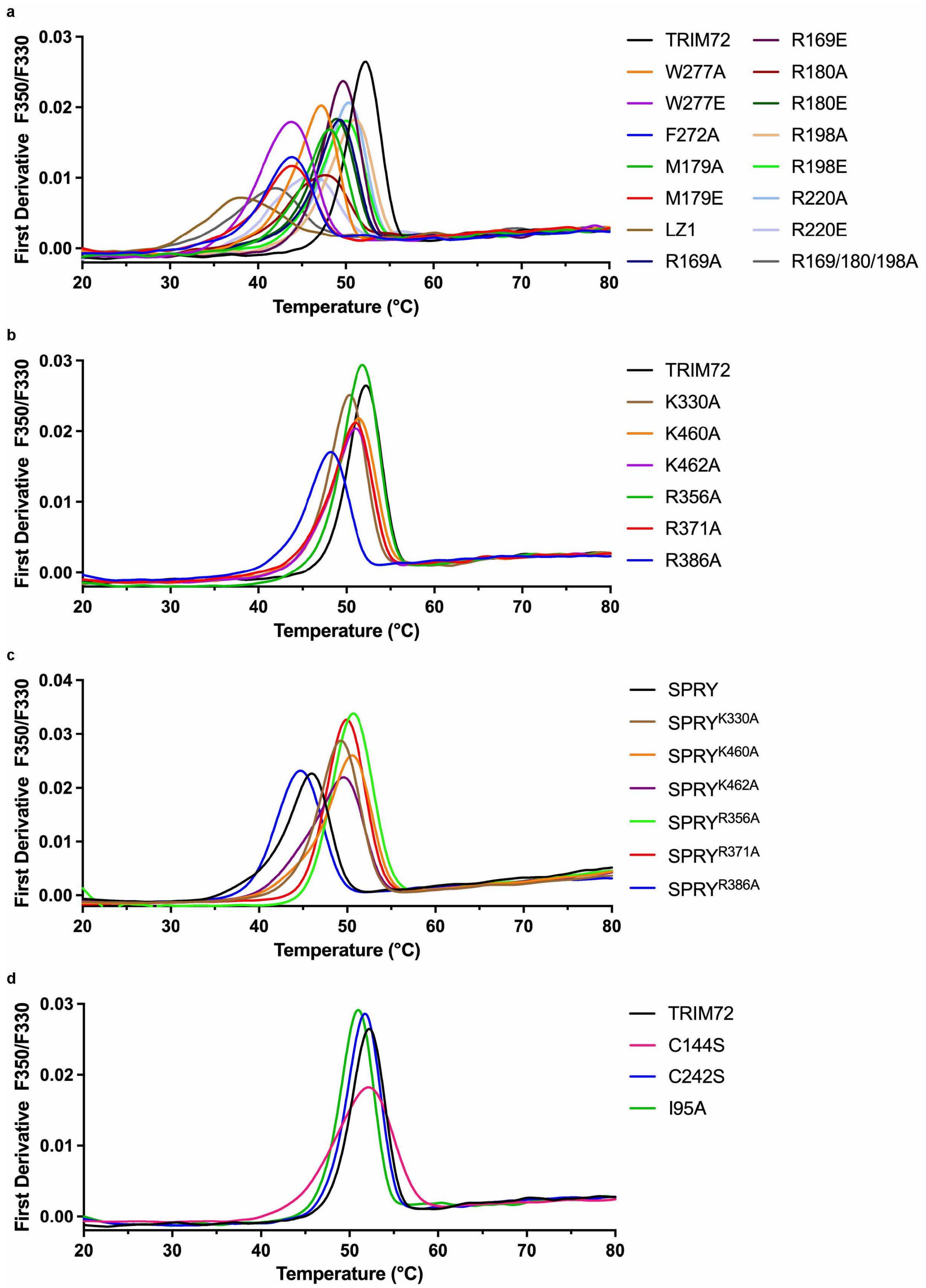

Supplementary Fig. 8

(a-d) The melting curves of TRIM72 WT and mutants in nano-DSF thermal shift assays, related to Fig. **2g, 4f, 5j**. Data are representative of two experiments.

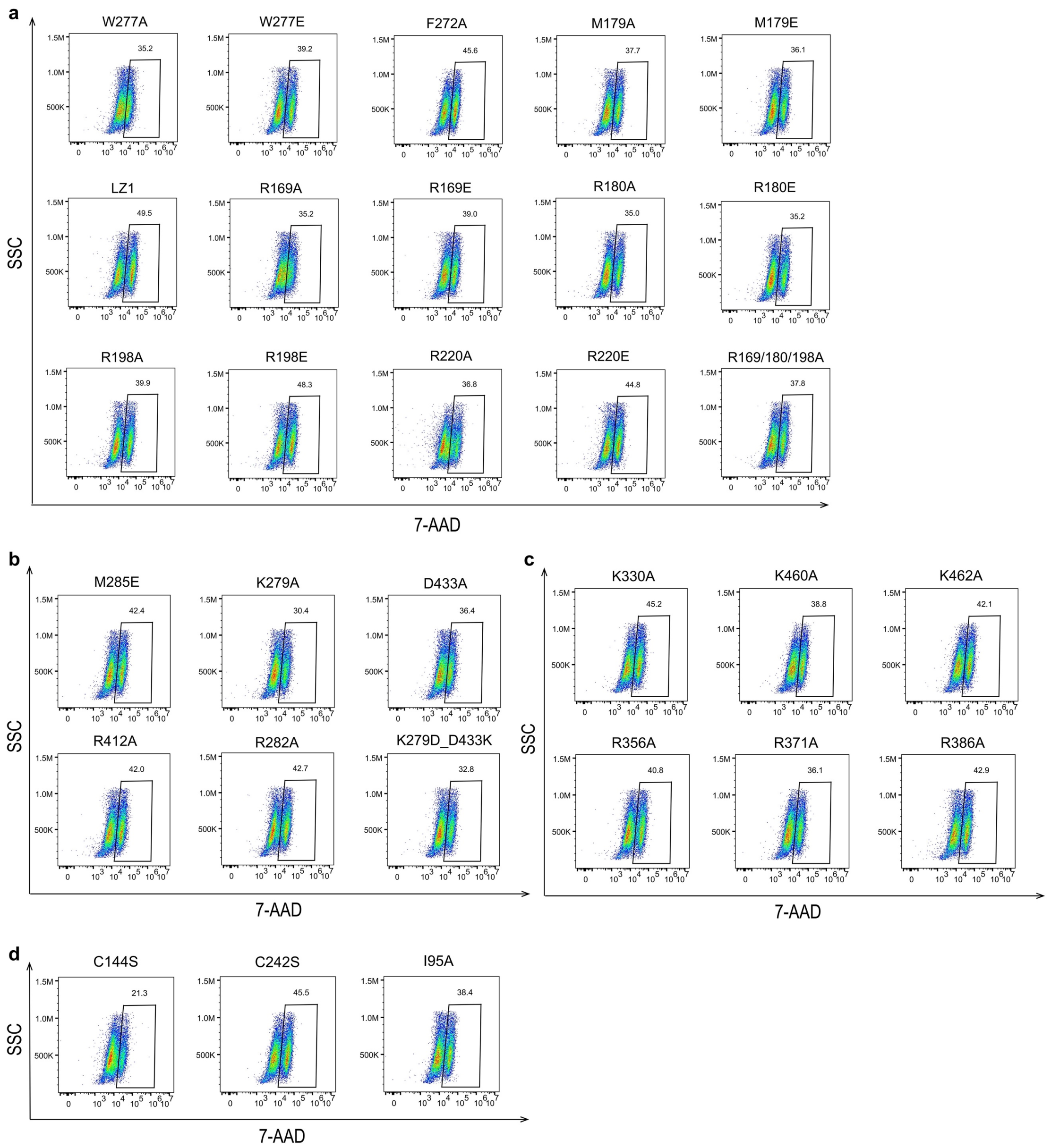

Supplementary Fig. 9

(a-d) Cell viability analysis of HEK293T cells overexpressing various TRIM72 constructs after H_2O_2/saponin treatment, related to Fig. **2h, 3c, 4g, 5i**. Cells were stained by 7-AAD and analyzed with flow cytometry, representative images of three independent experiments are shown.

a

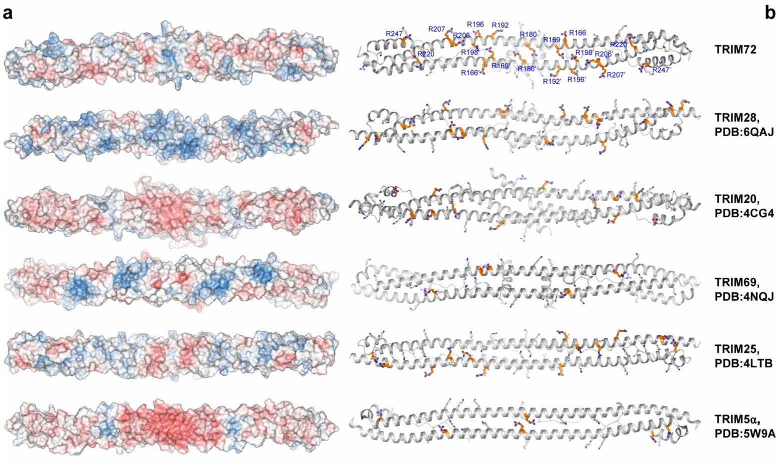

b

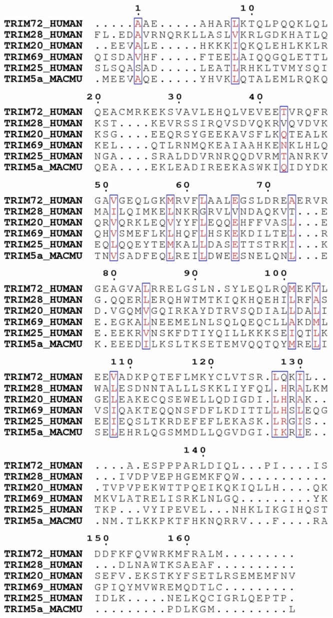

c

	TRIM72		TRIM28		TRIM20		TRIM69		TRIM25		TRIM5α	
Amino Acid	Lys	Arg	Lys	Arg	Lys	Arg	Lys	Arg	Lys	Arg	Lys	Arg
Number	11	15	17	12	20	8	14	5	19	11	15	9
Percentage(%)	6.71	9.15	10.49	7.41	11.43	4.57	8.33	2.98	11.11	6.43	9.68	5.81

Supplementary Fig. 10

(**a**) Electrostatic surface potentials of the coiled-coil domain from six TRIM family proteins, positive and negative potentials are colored blue and red, respectively. Positively charged arginine and lysine residues are displayed as sticks, arginine residues on the front-facing surface of the coiled-coil domains are colored in orange.

(**b**) Sequence alignment of coiled-coil domains from human TRIM72, TRIM28, TRIM20, TRIM69, TRIM25, and rhesus macaque TRIM5α.

(**c**) The number and percentage of lysine or arginine residues in the coiled-coil domains are listed.

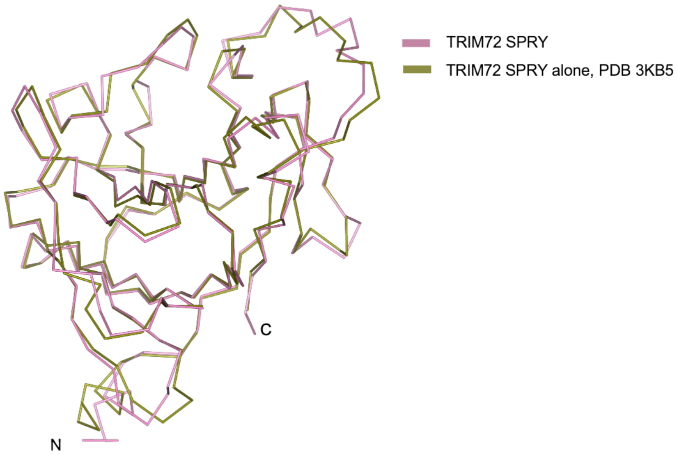

Supplementary Fig. 11

Superposition of TRIM72 SPRY alone structure (PDB: 3KB5) to the SPRY region from TRIM72 BCC-SPRY structure.

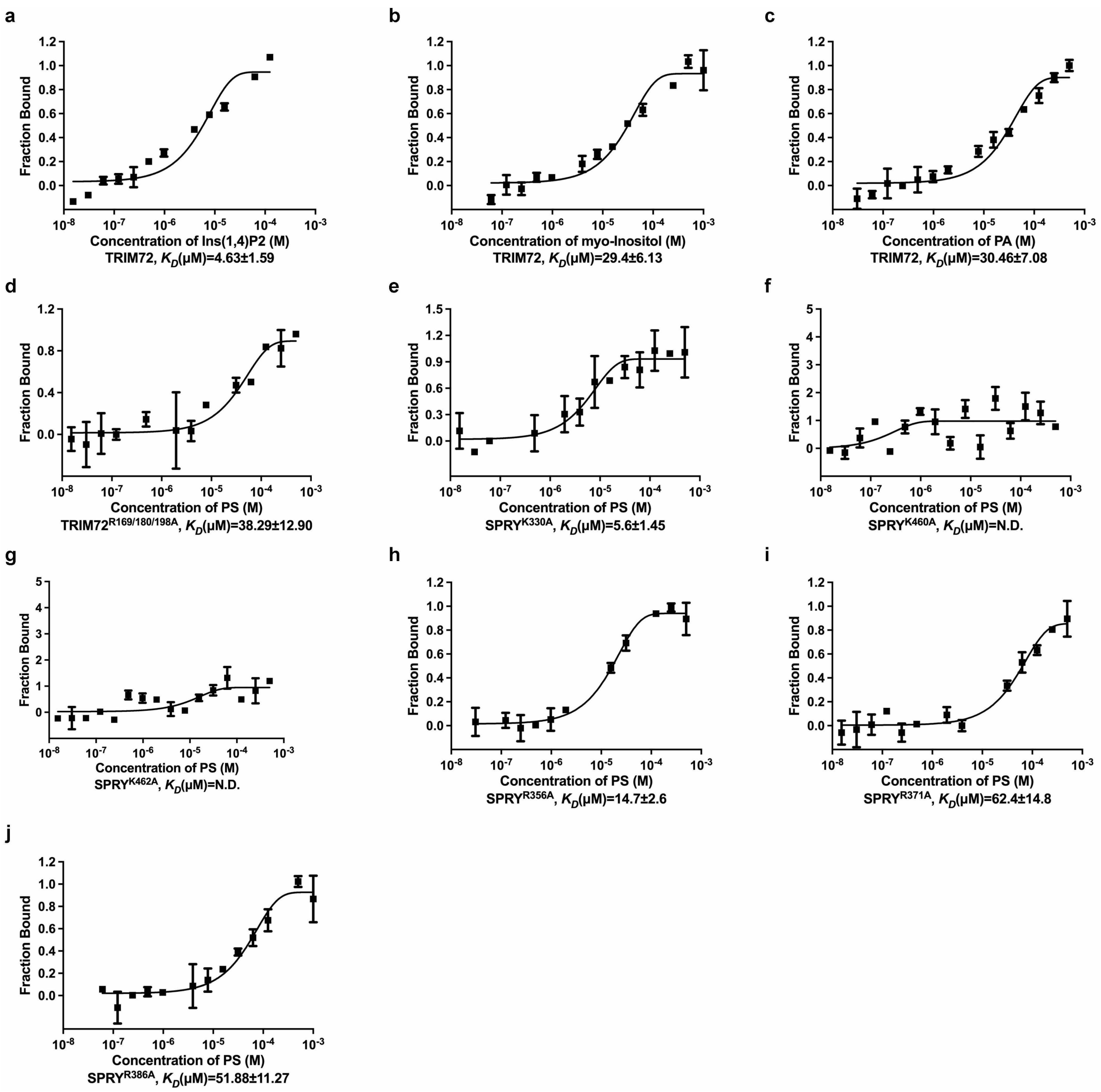

Supplementary Fig. 12

(**a**-**c**) MST titration results of TRIM72 with inositol 1,4-bisphosphate (Ins(1,4)P2, **a**), myo-inositol (**b**) or 1,2-dihexanoyl-sn-glycero-3-phosphate (PA, **c**),

(**d**) MST titration results of $TRIM72^{R169/180/198A}$ with phospho-L-serine (PS).

(e-j) MST titration results of SPRY domain (278-477) mutants with phospho-L-serine (PS). Data represent mean ± SEM from n = 3 independent experiments. Source data are provided as a Source Data file.

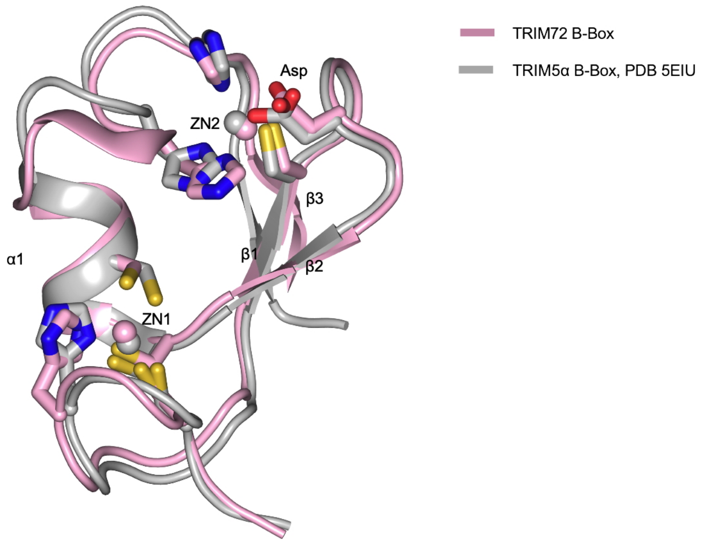

Supplementary Fig. 13

Superposition of the TRIM5α B-box with TRIM72 B-box yielded an RMSD of 0.81 Å over 32 aligned Cα atoms. Both proteins employ a conserved Asp residue to coordinate ZN2.

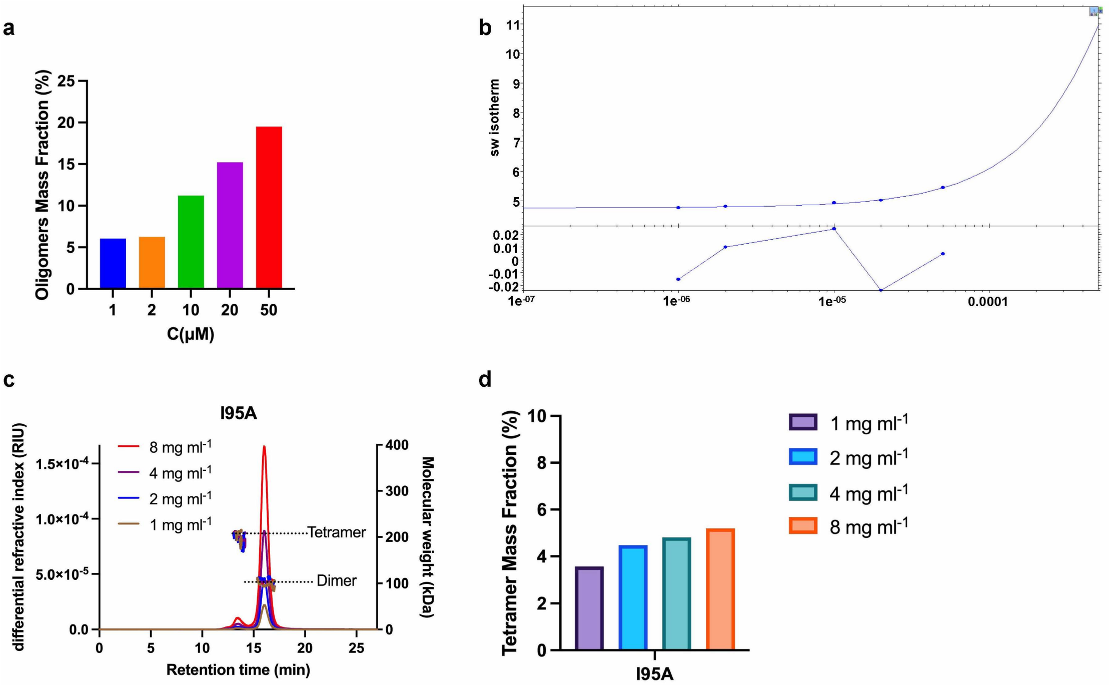

Supplementary Fig. 14

(**a**-**b**) The percentage of oligomer mass fraction of TRIM72 I95A in SV-AUC at various protein concentrations. Signal weighted-average sedimentation coefficient (sw) isotherm analysis in SEDPHAT is shown in **b**. The KD for TRIM72 I95A tetramer formation was calculated to be around 2.2 mM.

(**c**-**d**) SEC-MALS analysis of TRIM72 I95A at a range of protein concentrations. The percentage of tetramer mass fraction of TRIM72 is shown in **d**.

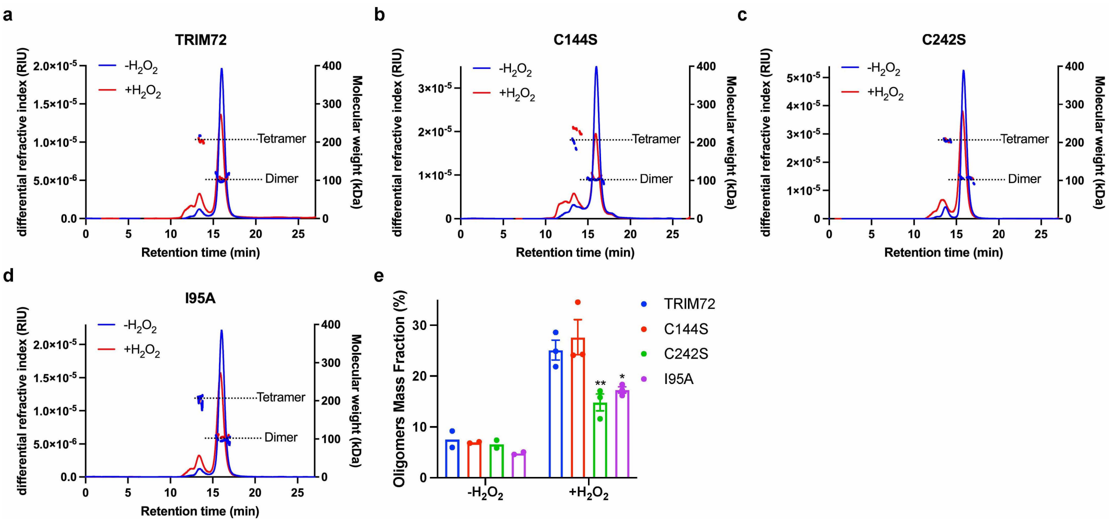

Supplementary Fig. 15

(**a**-**d**) SEC-MALS analysis of TRIM72 WT, C144S, C242A, or I95A mutant proteins (1 mg/mL) before or after incubation with 0.04% H_2O_2.

(**e**) The percentages of oligomer mass fraction are shown, data are from three independent experiments, mean ± SEM; *$p < 0.05$, **$p < 0.01$; one-way ANOVA with Dunnett´s multiple comparison test. +H_2O_2 (C144S: p =0.9553; C242S: p = 0.0034; I95A: p = 0.0143).

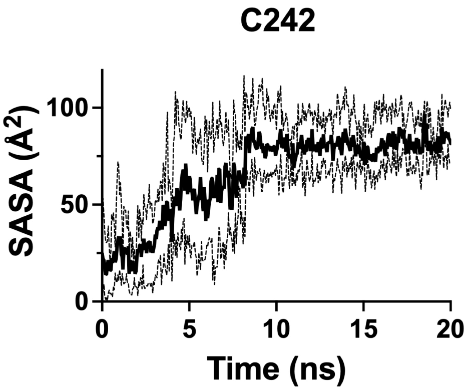

Supplementary Fig. 16

Solvent-accessible surface area (SASA) of Cys242 increased from around 20 Å^2 to a plateau of ~70 Å^2 during steered molecular dynamics (sMD) simulations after force application. The solid line indicates the mean SASA of four independent simulations, and the dashed lines indicate the SASA range of four independent simulations.

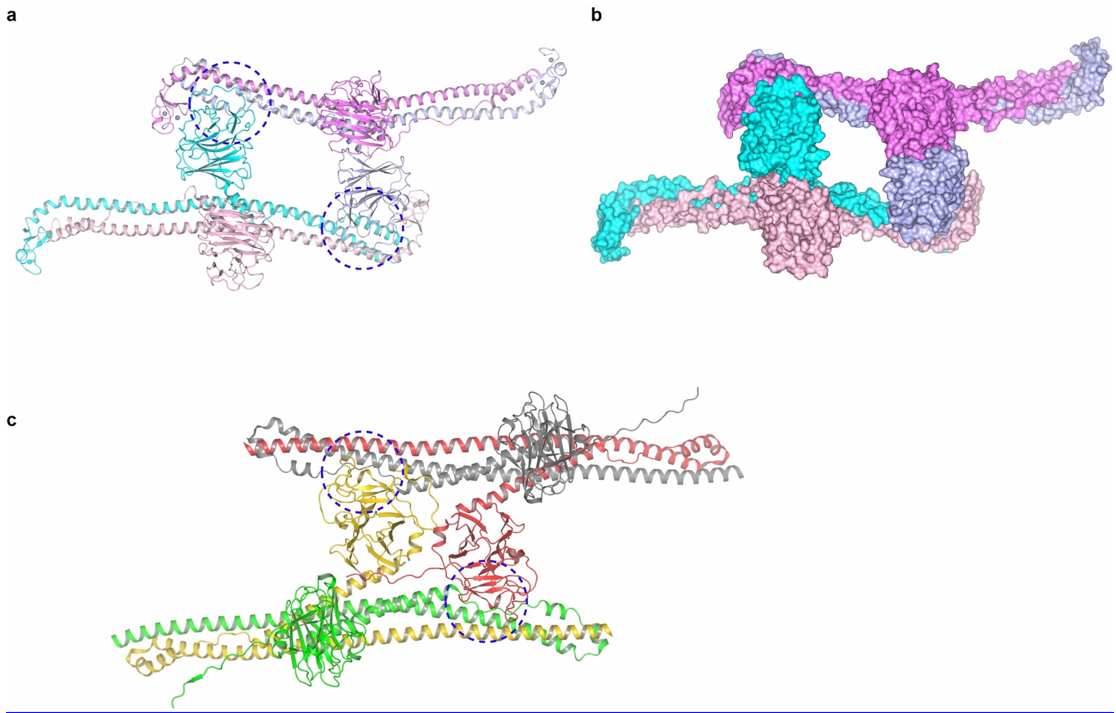

Supplementary Fig. 17

Side-by-side packing of TRIM72 and TRIM20.

(**a**) Ribbon presentation of two nearby TRIM72 BCC-SPRY dimers in the crystal lattice, SPRY contacts the three-helix bundle region of the coiled-coil domains (circled areas).

(**b**) Surface representation of **a**.

(**c**) Ribbon representation of two TRIM20 CC-SPRY dimers (PDB: 4CG4), SPRY of TRIM20 interacts with the proline-rich stretch in the coiled-coil region, circled areas are the contact interfaces.

Supplementary Table 1. Data collection and refinement statistics

**Data Set**	**TRIM72**
Space group	*C2*
a, b, c (Å)	72.57, 125.23, 174.54
α, β, γ (°)	90.0, 91.45, 90.0
Resolution (Å)	43.1 - 3.0 (3.11 – 3.00)
R_{sym}	0.097 (2.5)
R_{pim}	0.105 (2.7)
$I/\sigma(I)$	13.14 (0.75)
$CC^{1/2}$	0.999 (0.51)
Completeness (%)	98.6 (99.0)
Redundancy	6.9 (7.1)
**Refinement**	
Resolution (Å)	41.1 - 3.0
No. of reflections	30,857
R_{work}/R_{free} (%)	22.1/25.5
Protein atoms	6,138
Ligands	4
Solvent atoms	0
B factors (Å2)	140.2
Rmsd bond lengths (Å)	0.012
Rmsd bond angles (°)	1.39
Ramachandran outliers (%):	0
Ramachandran favored (%):	96.23

Values in parentheses are for the highest-resolution shell.
