# Supplementary material for: Structural basis for TRIM72 oligomerization during membrane damage repair
Source: Nat Commun. 2023 Mar 21;14:1555. doi: 10.1038/s41467-023-37198-1 (PMC10030467; doi:10.1038/s41467-023-37198-1)
Supplement: Supplementary file 2 — Description of Additional Supplementary Files [file 41467_2023_37198_MOESM2_ESM.pdf]

Description of Additional Supplementary Files

File Name: Supplementary Data 1
Description: Coordinates of TRIM72 from MD simulation experiments, showing the initial configuration.

File Name: Supplementary Data 2
Description: Coordinates of TRIM72 from MD simulation experiments, showing the final configuration.

File Name: Supplementary Data 3
Description: Excel file containing sequences of primers used in this study.

File Name: Supplementary Movie 1
Description: Molecular dynamics simulation trajectories showing the exposure of C242 after a force has been applied to the B-box, TRIM72 chain A is colored in wheat, chain B is colored in lightgreen, C242 is shown as red spheres. Related to Supplementary Fig. 16
