# Supplementary material for: Structural basis for TRIM72 oligomerization during membrane damage repair
Source: Nat Commun. 2023 Mar 21;14:1555. doi: 10.1038/s41467-023-37198-1 (PMC10030467; doi:10.1038/s41467-023-37198-1)
Supplement: Supplementary file 7 — Reporting Summary [file 41467_2023_37198_MOESM7_ESM.pdf]

nature portfolio

Corresponding author(s): Chun Zhou

Last updated by author(s): Feb 7, 2023

Reporting Summary

Nature Portfolio wishes to improve the reproducibility of the work that we publish. This form provides structure for consistency and transparency in reporting. For further information on Nature Portfolio policies, see our Editorial Policies and the Editorial Policy Checklist.

Statistics

For all statistical analyses, confirm that the following items are present in the figure legend, table legend, main text, or Methods section.

n/a	Confirmed	
☐	☒	The exact sample size (*n*) for each experimental group/condition, given as a discrete number and unit of measurement
☐	☒	A statement on whether measurements were taken from distinct samples or whether the same sample was measured repeatedly
☐	☒	The statistical test(s) used AND whether they are one- or two-sided<br>*Only common tests should be described solely by name; describe more complex techniques in the Methods section.*
☒	☐	A description of all covariates tested
☐	☒	A description of any assumptions or corrections, such as tests of normality and adjustment for multiple comparisons
☐	☒	A full description of the statistical parameters including central tendency (e.g. means) or other basic estimates (e.g. regression coefficient) AND variation (e.g. standard deviation) or associated estimates of uncertainty (e.g. confidence intervals)
☐	☒	For null hypothesis testing, the test statistic (e.g. *F*, *t*, *r*) with confidence intervals, effect sizes, degrees of freedom and *P* value noted<br>*Give P values as exact values whenever suitable.*
☒	☐	For Bayesian analysis, information on the choice of priors and Markov chain Monte Carlo settings
☒	☐	For hierarchical and complex designs, identification of the appropriate level for tests and full reporting of outcomes
☒	☐	Estimates of effect sizes (e.g. Cohen's *d*, Pearson's *r*), indicating how they were calculated

Our web collection on statistics for biologists contains articles on many of the points above.

Software and code

Policy information about availability of computer code

Data collection	CytoFlex flow cytometer (Beckman Coulter); confocal microscope(Nikon C2 confocal microscope);chemiluminescence imaging system (Clinx Science); DSF experiment was performed using Prometheus NT.48 nano-DSF (Nanotemper Technologies);MST experiment was performed using Monolith NT.115(Nanotemper Technologies); SV-AUC using Optima XL-I analytical ultracentrifuge (Beckman); SEC-MALS using a Wyatt HELEOS-II 18-angle photometer coupled to a Wyatt Optilab rEX differential refractometer (Wyatt Technology Corp);X-ray diffraction data were collected at SSRF BL19U1
Data analysis	XDSGUI (built 20201202); CCP4 (v7.1); Phenix (v1.20rc1_4392); Coot (v0.9.4.1), Pymol (v2.3.4); VMD (v1.9.3); GROMACS (v2022.3);CytExpert (v1.0); NIS-Elements imaging software (v5.01); Volocity(v6.1.1); GraphPad Prism (v9.0); FlowJo (v9.3.1); MO. Affinity Analysis software(v2.3); PR.ThermControl(v2.1.6); ASTRA (v6.1);SEDNTERP (http://www.rasmb.org/sednterp/);SEDFIT and SEDPHAT (sedfitsedphat.nibib.nih.gov/software)

For manuscripts utilizing custom algorithms or software that are central to the research but not yet described in published literature, software must be made available to editors and reviewers. We strongly encourage code deposition in a community repository (e.g. GitHub). See the Nature Portfolio guidelines for submitting code & software for further information.

Data

Policy information about availability of data

All manuscripts must include a data availability statement. This statement should provide the following information, where applicable:

- Accession codes, unique identifiers, or web links for publicly available datasets
- A description of any restrictions on data availability
- For clinical datasets or third party data, please ensure that the statement adheres to our policy

Coordinates and structure factors are available in the RCSB Protein Data Bank with accession code 7XT2 for TRIM72, other PDB entries used in this study: 4CG4 (TRIM20), 3KB5 (TRIM72 SPRY), 6QAJ (TRIM28), 4NQJ (TRIM69), 4LTB (TRIM25), 5W9A (TRIM5α) and 5EIU (TRIM5α B-Box). All other data are available in the main text or supplementary information. Source data are provided with this paper.

Human research participants

Policy information about studies involving human research participants and Sex and Gender in Research.

Reporting on sex and gender	N/A
Population characteristics	N/A
Recruitment	N/A
Ethics oversight	N/A

Note that full information on the approval of the study protocol must also be provided in the manuscript.

Field-specific reporting

Please select the one below that is the best fit for your research. If you are not sure, read the appropriate sections before making your selection.

☒ Life sciences ☐ Behavioural & social sciences ☐ Ecological, evolutionary & environmental sciences

For a reference copy of the document with all sections, see nature.com/documents/nr-reporting-summary-flat.pdf

Life sciences study design

All studies must disclose on these points even when the disclosure is negative.

Sample size	No statistical method was used to predetermine the sample size. Flow cytometry cell numbers were greater than 20000 for each construct, confocal imaging sample size was 5 cells for each construct; sample sizes were chosen based on well established protocols in the field.
Data exclusions	No data was excluded.
Replication	Biochemical experiments were performed at least twice and mostly three times to ensure reproducibility, all attempts of replication were successful and the data are provided in the Source Data file.
Randomization	Samples were not randomly allocated to groups as the experiments were independent replicates, randomization was not applicable.
Blinding	Blinding was not necessary for the experiments carried out in this study as investigator bias would not affect the results.

Reporting for specific materials, systems and methods

We require information from authors about some types of materials, experimental systems and methods used in many studies. Here, indicate whether each material, system or method listed is relevant to your study. If you are not sure if a list item applies to your research, read the appropriate section before selecting a response.

Materials & experimental systems

n/a	Involved in the study
☐	☒ Antibodies
☐	☒ Eukaryotic cell lines
☒	☐ Palaeontology and archaeology
☒	☐ Animals and other organisms
☒	☐ Clinical data
☒	☐ Dual use research of concern

Methods

n/a	Involved in the study
☒	☐ ChIP-seq
☐	☒ Flow cytometry
☒	☐ MRI-based neuroimaging

Antibodies

Antibodies used	1:1000 rabbit anti-FLAG antibody(Cell Signaling Technology, cat#14793), 1:5000 HRP-labeled goat anti-rabbit antibodies (Beyotime Biotechnology, cat#A0208).
Validation	All antibody validations are available on the manufacturers' websites.

Eukaryotic cell lines

Policy information about cell lines and Sex and Gender in Research

Cell line source(s)	C2C12 (SCSP-505, Cell bank of the Chinese Academy of Sciences),HEK293T (CRL-3216, ATCC)
Authentication	STR profiling was used for authentication by Cell bank of the Chinese Academy of Sciences and ATCC
Mycoplasma contamination	Negative as shown by Hoechst staining
Commonly misidentified lines (See ICLAC register)	No commonly misidentified cell lines were used

Flow Cytometry

Plots

Confirm that:

☒ The axis labels state the marker and fluorochrome used (e.g. CD4-FITC).

☒ The axis scales are clearly visible. Include numbers along axes only for bottom left plot of group (a 'group' is an analysis of identical markers).

☒ All plots are contour plots with outliers or pseudocolor plots.

☒ A numerical value for number of cells or percentage (with statistics) is provided.

Methodology

Sample preparation	After the cells were treated with H2O2 and saponin, they were centrifuged at 100xg for 5min, washed with PBS and resuspended in 300 ul PBS. Afterwards 5 ul 7-AAD was added to the cells and incubated on ice for 5 min, then the samples were analyzed with a CytoFlex flow cytometer.
Instrument	CytoFlex flow cytometer
Software	CytExpert 1.0, FlowJo 9.3.1
Cell population abundance	Debris and multiplet cells were eliminated using forward and side scatter parameters, 20000 cells were counted. Since we're examining cell viability, only 7-AAD was added to distinguish live and dead cells. The percentage of 7-AAD positive cells varied between 10% to 60%.
Gating strategy	Gating for 7AAD positive cells was based on a control sample without adding 7-AAD.

☒ Tick this box to confirm that a figure exemplifying the gating strategy is provided in the Supplementary Information.
